# Prevalence of sleep disorders among older adults in Chinese older adults care institutions: a systematic review and meta-analysis

**DOI:** 10.3389/fpubh.2025.1664136

**Published:** 2025-12-03

**Authors:** Xiaodi Bai, Shulan Liu, Yanxin Lv, Yuling Luo, Yunlan Jiang

**Affiliations:** 1 School of Nursing, Chengdu University of Traditional Chinese Mdeicine, Chengdu, China; 2 Hospital of Chengdu University of Traditional Chinese Medicine, Chengdu, China

**Keywords:** China, older adults care institutions, aged, sleep disorders, prevalence, meta-analysis

## Abstract

**Objective:**

To systematically evaluate the prevalence characteristics of sleep disorders among older adults residents in Chinese nursing homes and the differences among various subpopulations, providing evidence for promoting healthy aging.

**Methods:**

Search formulas were developed to systematically retrieve literature from the CNKI, VIP, Wan fang Data, CBM, PubMed, Web of Science, and Cochrane Library databases. Cross-sectional studies published from the inception of each database until May 2025 on the incidence of sleep disorders among older adults individuals in Chinese nursing homes were collected. Meta-analysis was performed using Stata 15.1 and R language (version 4.3.2).

**R**esults**:**

A total of 35 articles were finally included, involving 15,996 older adults individuals residing in nursing homes. Meta-analysis revealed a pooled detection rate of sleep disorders of 43% (95% CI: 38–48%) among this population. Temporal trend analysis indicated significant fluctuations in the incidence of sleep disorders from 2008 to 2024, coupled with an overall downward trend. Subgroup analyses revealed statistically significant differences (*p* < 0.05) based on geographic region, sample size, gender, and the sleep disorder assessment tool used.

**Conclusion:**

The detection rate of sleep disorders among older adults residents in Chinese nursing homes is relatively high, with marked disparities across different groups. Significant attention should be directed toward the sleep health of this population. Comprehensive preventive and intervention measures tailored to the characteristics of different subpopulations should be developed and implemented to effectively improve sleep quality and reduce the risk of sleep disorders.

**Systematic review registration:**

https://crd.york.ac.uk/prospero/, identifier (CRD420251056822).

## Introduction

With the accelerating pace of population aging, the demand for older adults care is growing rapidly. According to World Health Organization estimates, by 2050, 60% of the older adults population in China will require daily care and assistance (1). Consequently, the number of older adults choosing to reside in nursing homes is steadily increasing (2). Among the health-related issues they face, sleep problems are becoming particularly prominent.

Sleep health is a crucial component of the “Healthy China Action (2019–2030)” plan (3). Sleep disorders refer to conditions characterized by frequent and persistent difficulties in falling asleep and/or maintaining sleep, leading to dissatisfaction with sleep quality (4). Studies indicate that the overall detection rate of sleep disorders among older adults in China is approximately 46% (5, 6). Globally, the reported detection rate of sleep disorders among older adults residents in nursing homes is even higher, reaching about 65% (7). Adequate sleep is essential not only for maintaining normal physiological functions, such as immune system regulation and metabolic balance, but also significantly impacts mental health, including emotional stability and cognitive function preservation (8). However, sleep disorders not only significantly reduce the quality of life for older adults but also substantially increase their risk of falls (9) and limitations in activities of daily living (10), thereby further increasing the burden on both individuals and society.

Older adults residents in nursing homes often present unique characteristics, including older age, higher rates of disability, more complex comorbidities, and distinct psychosocial environments. Consequently, the mechanisms underlying sleep disorders and their epidemiological patterns may differ from those observed in community-dwelling or hospitalized populations. Large-scale, multi-center epidemiological studies focusing specifically on sleep disorders among older adults in Chinese nursing homes remain relatively scarce, and existing survey results exhibit a degree of heterogeneity.

Therefore, a systematic evaluation and synthesis of existing research are necessary to clarify the prevalence and trends of sleep disorders among older adults in Chinese nursing homes. This will provide an evidence-based foundation for healthcare professionals to enhance awareness and develop targeted prevention and intervention strategies. Simultaneously, it will offer scientific support for nursing home policy-makers in optimizing resource allocation, promoting the transition toward precision management of chronic diseases and care models for the older adults, and advancing from the goal of “healthy aging” to “active aging.”

## Materials and methods

This systematic review was performed based on the Preferred Reporting Items for Systematic Reviews and Meta-analyses Statement (PRISMA) guidelines and the detailed study protocol was registered on PROSPERO (CRD420251056822).

### Inclusion and exclusion criteria

#### Inclusion criteria

(1) Study type: Observational studies, including cross-sectional studies, case–control studies, and cohort studies; (2) Participants: Older adults individuals aged ≥60 years residing in nursing homes. Outcome; (3) Measures: Reported the incidence/prevalence of sleep disorders among older adults residents in nursing homes or provided data sufficient for calculation. Sleep disorders were defined using validated instruments or criteria such as the Pittsburgh Sleep Quality Index (PSQI), Athens Insomnia Scale (AIS), Insomnia Severity Index (ISI), Sleep Disorders Rating Scale (SDRS), or the International Classification of Sleep Disorders, Third Edition (ICSD-3). Publication; (4) Type: Peer-reviewed journal articles.

#### Exclusion criteria

(1) Duplicate publications; (2) Studies from which required data could not be extracted or that provided incomplete information; (3) Studies published in languages other than Chinese or English; (4) Reviews, conference abstracts, and proceedings. (5) Studies assessed as having critically low quality.

### Literature search strategy

Computerized searches will be conducted across the following databases: CNKI, WanFang Data, VIP, CBM, PubMed, Embase, Web of Science, and the Cochrane Library. The search will target studies reporting the prevalence/incidence of sleep disorders among older adults residents in nursing homes within China. The search strategy will combine Medical Subject Headings (MeSH)/Emtree terms and free-text keywords. To ensure comprehensiveness, supplementary manual searches of reference lists (backward citation tracking) and citation tracking (forward citation tracking) of included articles will be performed (“snowballing” method). The search timeframe will span from the inception of each database up to May 2025. Key search terms include: “China,” “Chinese”; “older adults homes,” “aged care homes,” “nursing homes”; “Sleep Wake Disorders,” “Sleep Disorders, Intrinsic,” “Dyssomnias,” “Sleep Disorders,” etc. The detailed search strategy for PubMed is provided as an example.

### Literature screening and data extraction

Literature screening will be managed using EndNote X9 software, utilizing its duplicate detection function for initial deduplication. Two reviewers, trained in systematic review methodology, will independently screen titles and abstracts against the predefined inclusion/exclusion criteria. Full texts of potentially eligible studies will then be retrieved and assessed independently. Any discrepancies during screening will be resolved through discussion with a third, senior reviewer. Data extraction will be performed using a standardized form, capturing the following information: first author, publication year, study design, study region/location, sample size, mean age, relevant outcome measures (e.g., prevalence/incidence of sleep disorders, diagnostic criteria/instrument used).

### Risk of bias assessment of included studies

The methodological quality of included cross-sectional studies will be assessed using the Agency for Healthcare Research and Quality (AHRQ) tool (11). Studies will be categorized as low quality (score 0–3), moderate quality (score 4–7), or high quality (score 8–11). The Newcastle-Ottawa Scale (NOS) (12) will be used to assess the risk of bias in included case–control and cohort studies. The NOS evaluates studies across three domains, with a maximum score of 9; studies scoring ≥6 will be considered high quality. Two reviewers will independently conduct the risk of bias assessments. Disagreements will be resolved through discussion or consultation with a third reviewer.

### Statistical analysis

Statistical analyses will be performed using Stata 15.0 and R software (version 4.3.2). For meta-analysis of proportions (prevalence/incidence), the raw proportions are transformed using the Freeman-Tukey double arcsine transformation method (13) to stabilize variances, especially when data are non-normally distributed or proportions are near 0 or 1. The primary pooled outcome will be the prevalence/incidence rate with its corresponding 95% confidence interval (*CI*). Heterogeneity among studies will be evaluated using the Cochran’s Q test and quantified using the *I*^2^ statistic. A fixed-effects model will be employed if heterogeneity is low (*p* > 0.1 for Q test and *I*^2^ < 50%); otherwise, a random-effects model will be used. Potential publication bias will be assessed visually using funnel plots and statistically using Egger’s test. *p* < 0.05 indicates a statistically significant publication bias. Sensitivity analyses will be conducted using the leave-one-out method to evaluate the robustness of the results. Alternatively, subgroup analyses based on characteristics such as geographic region, sample size, gender, assessment tool, duration of nursing home residence, and depressive status were conducted to explore the sources of heterogeneity.

## Results

### Literature screening process and results

**Figure 1:**
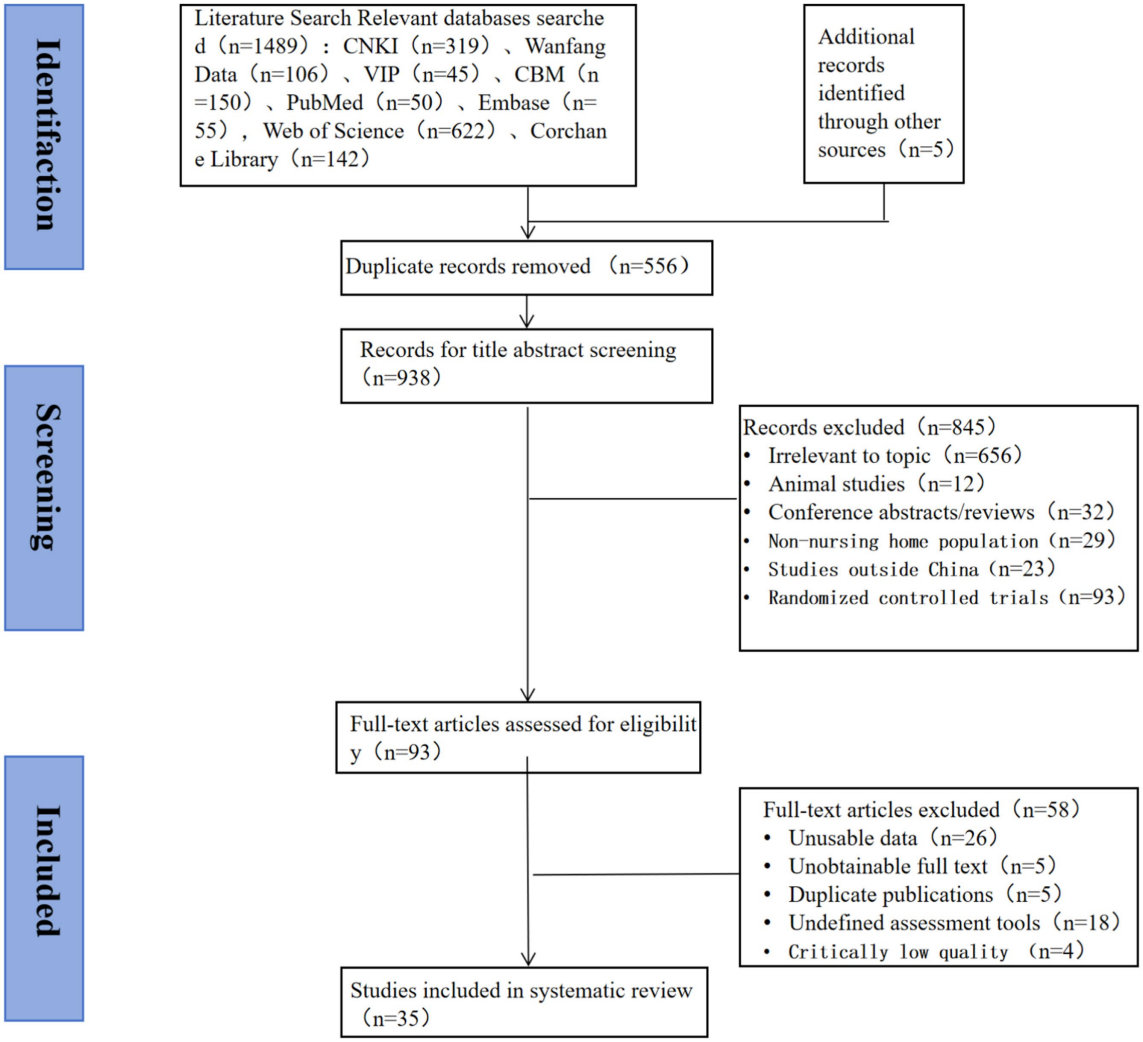
Flow chart for screening included literature.

A comprehensive search of relevant databases initially retrieved 1,494 records. Through a rigorous screening process, 35 studies ultimately met the inclusion criteria and were selectedfor this review. The flow diagram detailing the screening process is presented in Figure 1.

### Characteristics and quality assessment of included studies

A total of 35 studies were included in this systematic review. The quality assessment scores ranged from 4 to 9 points. Twenty-five studies were rated as moderate quality, and 10 studies were classified as high quality. These studies encompassed 17 provincial-level regions in China. Detailed characteristics are presented in Table 1.

**Table 1:** Basic characteristics of the included literature.

First author	Year	Region	Mean age	Questionnaire	Sample size (cases)	Sleep disorders (cases)	Prevalence rate	Quality assessment
Hao, 2023 (27)	2021–2023	Beijing	74.8 ± 8.4	PSQI>7	203	97	47.78	6
Han, 2023 (28)	-	Changchun	-	PSQI>7	411	201	48.91	7
Zhu, 2022 (29)	-	Hangzhou	80.80 ± 7.35	PSQI>7	402	160	39.8	7
Wu, 2016 (30)	2014–2015	Fuzhou	83.52 ± 6.97	PSQI>7	176	96	54.5	8
Gong, 2012 (31)	2010	Tangshan	79.29 ± 7.04	PSQI>7	409	211	51.59	4
Zhu, 2015 (32)	-	Chongqing	82.01 ± 8.22	PSQI>7	123	53	43.0	6
Huang, 2019 (33)	-	Shanghai	80.66 ± 7.61	PSQI>7	410	259	63.17	5
Yu, 2011 (34)	-	Siping	74.5 ± 8.76	PSQI>7	100	61	61.0	5
Liu, 2023 (35)	2022–2023	Weifang	79.84 ± 7.71	AIS>6	453	106	23.4	7
Wang, 2019 (36)	2017–2018	Nanjing	83.1 ± 6.0	AIS>6	516	222	43.0	8
Yang, 2021 (37)	2018–2019	Jinan	-	AIS>6	353	78	22.0	9
Wu, 2020 (38)	2016	Jinan	77.45 ± 9.05	AIS>6	296	112	38.1	9
Wang, 2024 (39)	-	Changchun	82.28	PSQI≥8	87	36	41.4	6
Cai, 2022 (40)	2020	Fuzhou	77.67 ± 8.39	PSQI≥5	275	189	68.73	8
Li, 2012 (41)	2011	Beijing	82.48 ± 6.85	PSQI>7	107	35	32.71	7
Gao, 2023 (42)	2021	Weifang	82.08 ± 8.13	PSQI≥11	381	135	35.43	8
Zhao, 2025 (43)	2019	Shanghai	-	ASI > 6	739	307	41.54	7
Xia, 2018 (44)	-	Wenzhou	-	PSQI>7	100	39	39.0	7
Chen, 2014 (45)	2012	Wuhan	-	PSQI>7	151	98	64.9	7
Wu, 2021 (46)	2019	Chengdu, Deyang	73.67 ± 7.75	PSQI>7	351	114	32.4	8
Nie, 2020 (47)	2018	Changsha, Hengyang, Yiyang	79.10 ± 8.71	PSQI>7	817	550	67.3	8
Zhang, 2020 (48)	2017	Shenyang, Anshan, Tieling, Benxi	71.9 ± 4.8	PSQI>7	553	254	31.1	7
Gao, 2023 (49)	2021–2022	Weifang	77.43 ± 9.96	PSQI≥11	645	214	33.2	7
Wang, 2022 (50)	2019–2020	Xuzhou	80.8 ± 6.3	PSQI>7	228	88	38.6	7
Liu, 2022 (51)	2021	Huaihua, Shaoyang Changde, Xiangtan	77.32 ± 8.87	PSQI>7	1,206	735	60.9	7

Hu, 2023 (52)	2020–2021	Guangzhou	70.56 ± 7.58	PSQI>7	259	136	52.5	7
Liu, 2019 (53)	-	Xining	-	PSQI>7	207	86	41.54	6
		Guangzhou	-	PSQI>7	437	82	18.76	
Jiang, 2019 (54)	-	Shanghai	84.33 ± 6.9	ASI>6	605	120	19.83	7
Sun, 2024 (55)	2023	Jilin, Liaoning Heilongjiang Provinces	76.79 ± 8.56	PSQI>5	574	127	22.1	7
Wang, 2021 (56)	2018	Shanghai	86.40 ± 5.27	AIS>6	218	56	25.7	7
Tsai, 2008 (57)	-	Taiwan Province	79.0 ± 6.7	PSQI>5	196	91	46.4	8
Wen, 2024 (58)	2021	Hunan Province	77.52 ± 9.16	PSQI>7	3,356	1,035	30.8	7
Hou, 2024 (59)	2021–2022	Changsha	-	PSQI>7	172	108	62.8	7
Zhu, 2024 (60)	2023	Chongqing	81.54 ± 7.94	PSQI>7	127	71	55.96	7
Mou, 2020 (61)	2018	Jinan	78.81 ± 8.90	PSQI>7	353	221	62.6	8

“-” Indicates that the item was not reported in the study. Assessment tools: Pittsburgh Sleep Quality Index (PSQI); Athens Insomnia Scale (AIS).

### Prevalence of sleep disorders in nursing home residents

**Figure 2:**
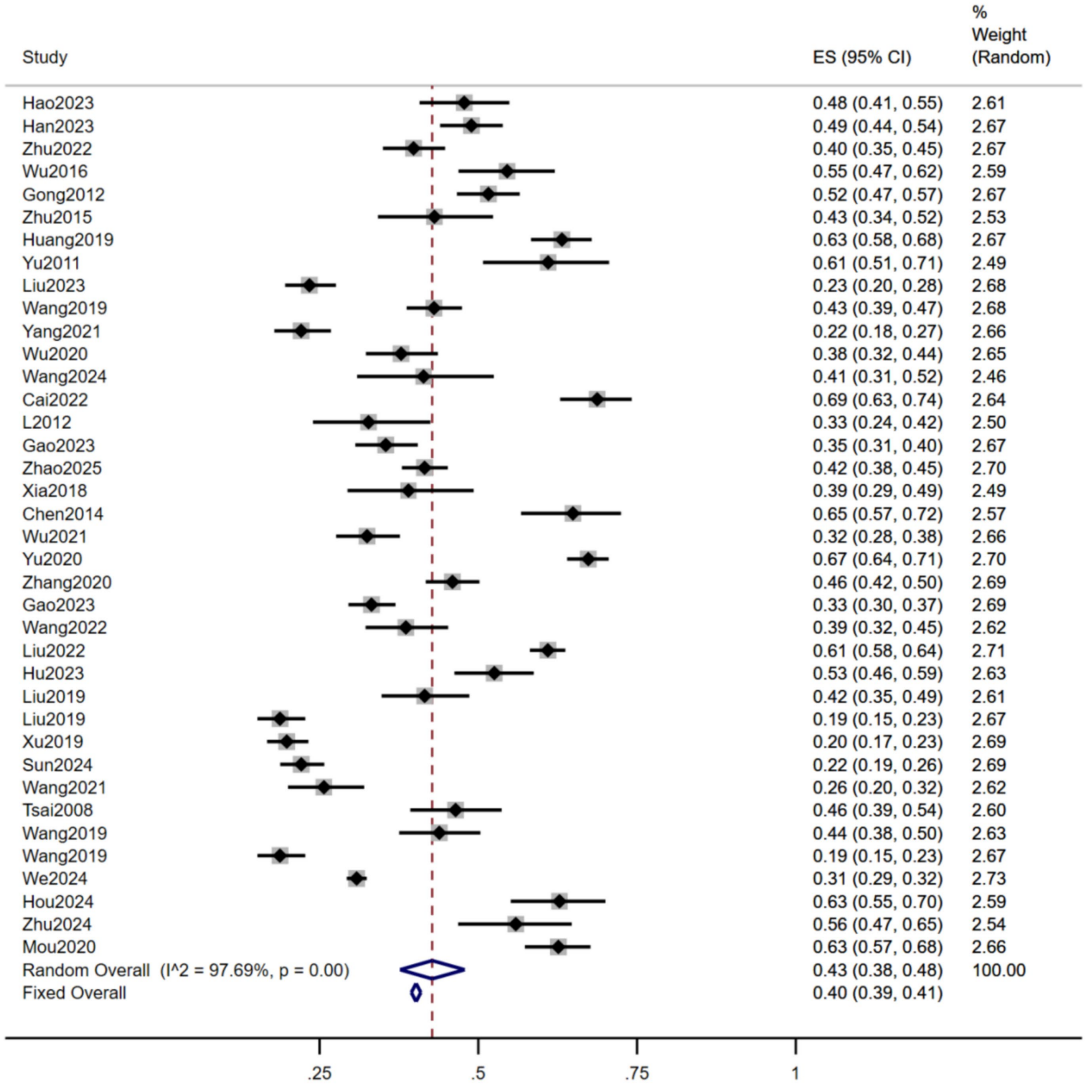
Forest plot of the prevalence of sleep disorders among older adults residents in nursing homes in China.

A total of 35 studies involving 15,996 older adults residents in nursing homes were included in this meta-analysis. Among them, 6,583 individuals were identified as having sleep disorders. Due to significant heterogeneity among the included studies, a random-effects model was employed to pool the effect sizes. The meta-analysis revealed that the pooled prevalence of sleep disorders among older adults residents in nursing homes in China was 43% (95% CI: 38 to 48%), as illustrated in Figure 2.

### Temporal trends in sleep disorder prevalence

**Figure 3:**
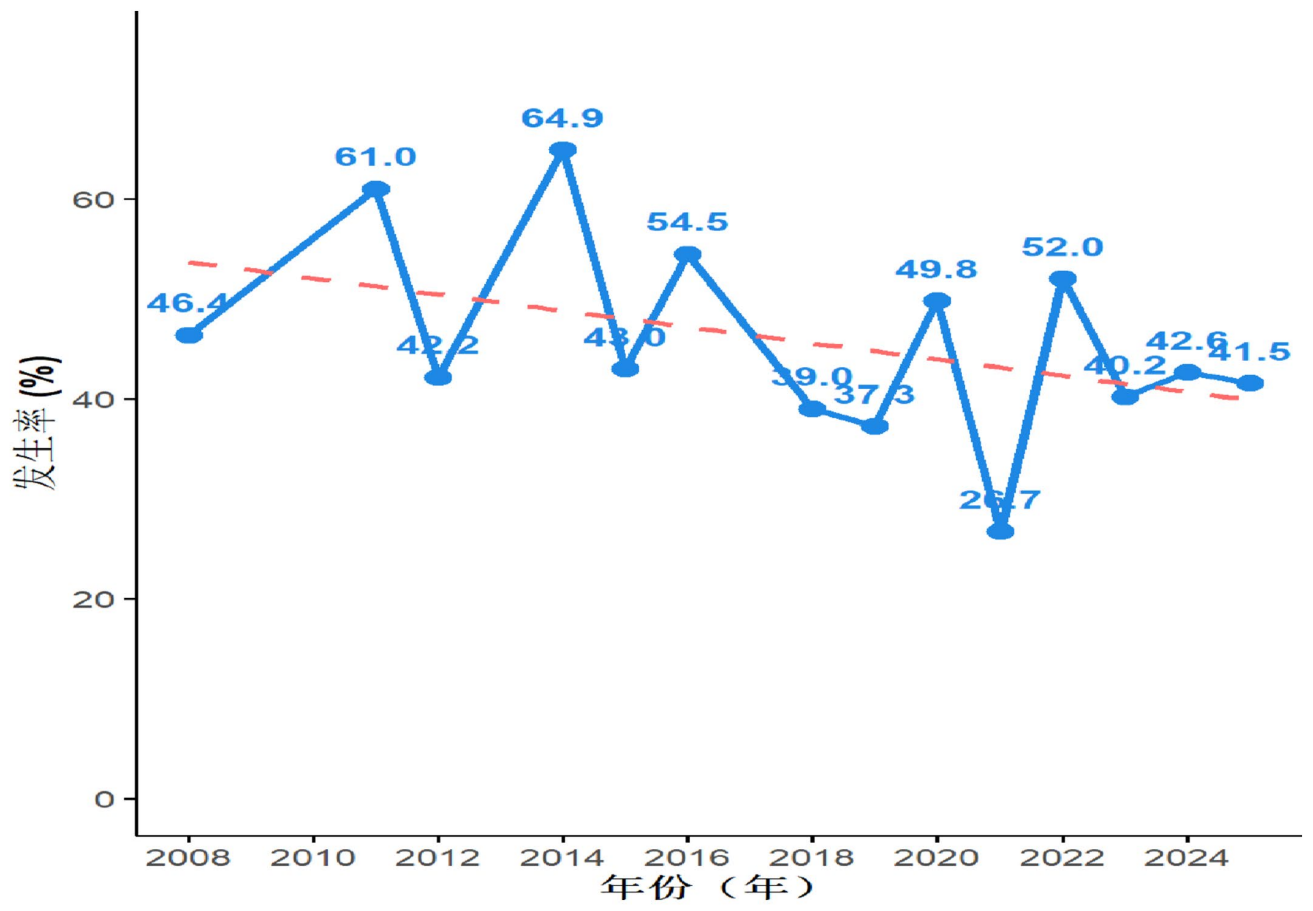
Temporal trend in the prevalence of sleep disorders among older adults residents in nursing homes in China (2008–2024).

Analysis of temporal trends demonstrated significant fluctuations in the prevalence of sleep disorders among nursing home residents in China between 2008 and 2024. Despite these fluctuations, a general downward trend was observed. Notably, a transient increase in prevalence occurred in 2022. The temporal trend is detailed in Figure 3.

### Subgroup analyses

#### Region

Studies were stratified according to China’s six major administrative regions. Meta-analysis results indicated the highest prevalence of sleep disorders in nursing homes was in Central China (57%), while the lowest prevalence was found in South China. The East China region contributed the largest number of included studies (*n* = 16), with a pooled prevalence of 40%. The Northwest China and Taiwan/Hong Kong/Macau regions were analyzed separately due to limited included studies (*n* = 1). Hence, these findings have limited reliability and generalizability and are not suitable for independent interpretation or extrapolation (See Table 2).

**Table 2:** Subgroup analysis of sleep disorder prevalence among older adults residents in nursing homes in China.

Subgroup	Included studies	Heterogeneity test		Effect model	Prevalence rate (95% CI)
		*I^2^*	*p*		
Region					
North China	3	85	0.01	Random	45% (34%, 55%)
Northeast China	5	97	0.01	Random	44% (31%, 56%)
East China	16	98	0.01	Random	40% (33%, 38%)
Central China	5	99	0.01	Random	57% (44%, 71%)
South China	2	99	0.01	Random	28% (25%, 31%)
Southwest China	3	91	0.01	Random	43% (30%, 57%)
Northwest China	1	-	-		42% (35%, 49%)
Taiwan/Hong Kong/Macao	1	-	-		46% (39%, 54%)
Sample size					
≤150	6	81	0.01	Random	46% (37%, 54%)
150–300	11	94	0.01	Random	49% (41%, 57%)
300–500	10	98	0.01	Random	40% (30%, 50%)
500–1,000	7	99	0.01	Random	39% (27%, 51%)
>1,000	2	100	0.01	Random	48% (37%, 40%)
Duration of residence					
>1个月	4	95	0.01	Random	42% (26%, 58%)
≥3个月	12	99	0.01	Random	41% (32%, 50%)
≥6个月	7	91	0.01	Random	44% (35%, 52%)
≥12个月	2	96	0.01	Random	56% (32%, 79%)
Diagnostic criteria					
PSQI>7	23	98	0.01	Random	49% (43%, 54%)
PSQI≥8	1	-	-	Random	41% (31%, 52%)
AIS>6	3	96	0.01	Random	30% (23%, 38%)
PSQI≥11	2	0	0.46	Fixed	34% (31%, 37%)
PSQI>5	3	99	0.01	Random	46% (19%, 72%)
Gender					
Male	7	96	0.01	Random	37% (27%, 46%)
Female	7	95	0.01	Random	41% (32%, 50%)
Depression status					
Yes	7	92	0.01	Random	53% (40%, 66%)
No	7	83	0.01	Random	23% (16%, 31%)

“-” Indicates that the item was not reported in the study.

#### Sample size

Subgroup analysis based on sample size revealed that the highest prevalence of sleep disorders (49%) was reported in studies with sample sizes between 150 and 300. Conversely, the lowest prevalence (39%) was observed in studies with sample sizes between 500 and 1,000. These findings suggest a potential influence of sample size on prevalence estimates (See Table 2).

#### Length of nursing home residence

Stratification by duration of residence in nursing homes showed that the highest prevalence of sleep disorders (56%) occurred among residents with a length of residence ≥12 months. In contrast, the lowest prevalence (41%) was found among those with a length of residence ≥3 months (See Table 2).

#### Diagnostic criteria for sleep disorders

Subgroup analysis according to the diagnostic criteria used demonstrated the highest prevalence (49%) when sleep disorders were defined using the criterion PSQI >7. The lowest prevalence (30%) was observed when the AIS >6 criterion was applied. The widest confidence interval (46%; 95% CI: 19 to 72%) was associated with the PSQI >5 criterion (See Table 2).

#### Gender

Gender-based subgroup analysis indicated a slightly higher prevalence of sleep disorders among female residents (41%) compared to male residents (37%). However, the magnitude of this gender difference was relatively small (See Table 2).

### Sensitivity analysis

Sensitivity analysis was performed using the leave-one-out method. After sequentially excluding individual studies, the estimated prevalence of sleep disorders among older adults residents in nursing homes in China ranged between 39 and 48%. The pooled prevalence remained stable throughout this process, indicating that the meta-analysis results are robust.

### Publication bias assessment

**Figure 4:**
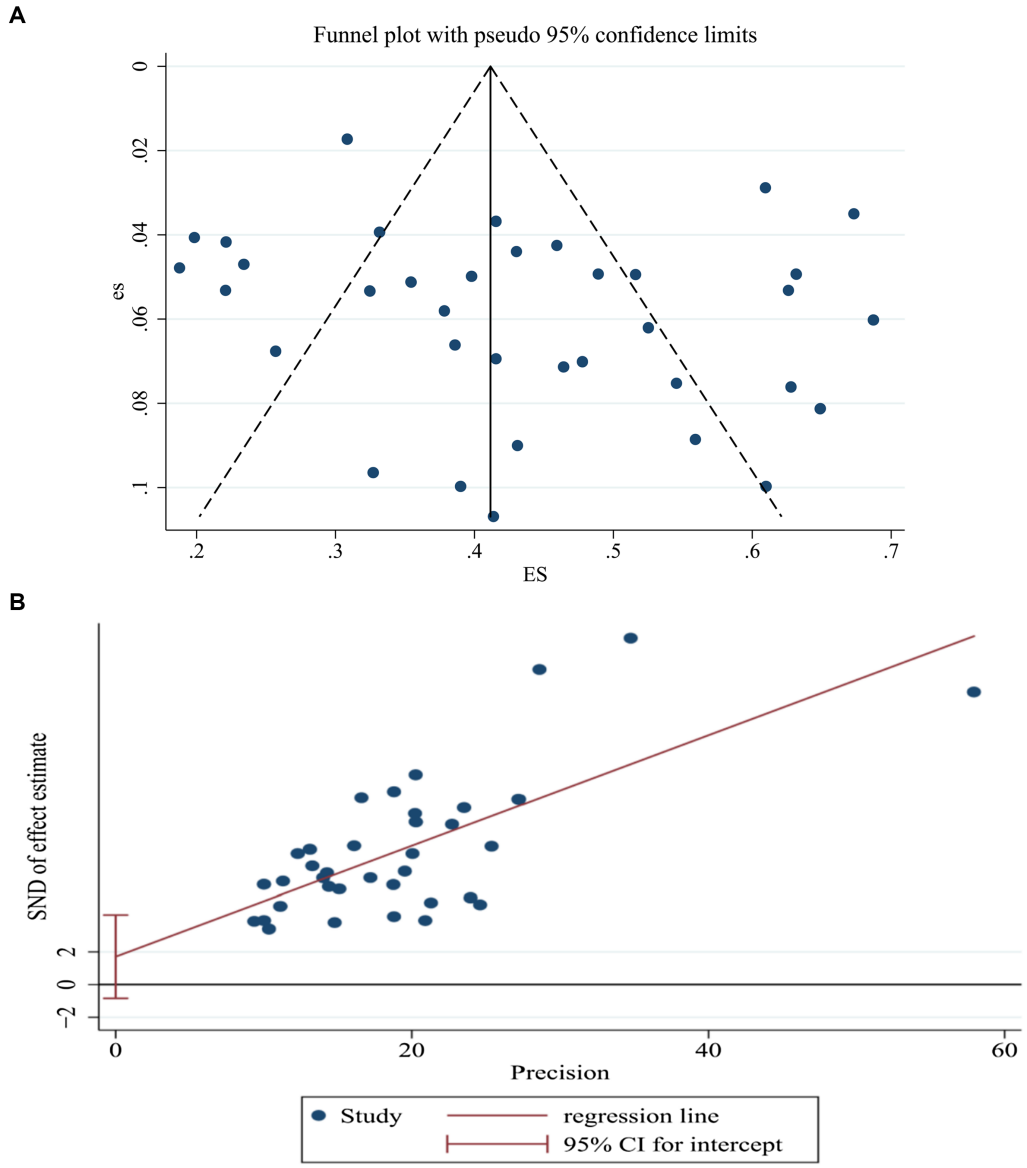
Assessment of publication bias for sleep disorder prevalence in nursing homes: **(A)** Funnel plot; **(B)** Egger’s regression test.

Publication bias was assessed for the included studies. The funnel plot for the prevalence of sleep disorders among nursing home residents demonstrated approximate symmetry (Figure 4). Egger’s linear regression test further confirmed no significant publication bias (*t* = 1.36, *p* = 0.182). These results suggest the absence of substantial publication bias in the current meta-analysis.

## Discussion

This systematic review incorporated 35 studies conducted between 2008 and 2024, encompassing 17 provinces (autonomous regions, and municipalities) in China. The results indicate that the prevalence of sleep disorders among older adults residents in Chinese nursing homes is 43%, which is marginally higher than that reported for community-dwelling older adults (41%). This finding aligns with international research indicating poorer sleep quality among residents of long-term care facilities compared to their community-dwelling counterparts (14, 15).

Our analysis revealed a significantly higher prevalence of sleep disorders among nursing home residents in Central China compared to other regions, with the lowest prevalence observed in South China. Prevalence rates in other regions showed minimal variation. This spatial heterogeneity may stem from several factors: the uneven geographical distribution of nursing homes (characterized by a higher concentration in eastern regions compared to western areas), regional disparities in socio-economic development levels, and imbalanced allocation of older adults care service resources (1). Research suggests a negative correlation between social support and sleep disorders in the older adults (16). Compared to community-dwelling seniors, nursing home residents often experience reduced opportunities for interaction with family members and neighbors, leading to a weakened social support network (17). This diminished social engagement reduces emotional support and participation, directly impairing sleep quality.

Our findings indicate that sample size did not significantly influence the estimated prevalence of sleep disorders in this population. Regarding diagnostic tool efficacy, the highest prevalence (49%) was observed when using the PSQI >7 cutoff, while the lowest prevalence (30%) was associated with the AIS > 6 criterion. This discrepancy likely arises from the multidimensional assessment advantage of the PSQI, which comprehensively evaluates seven domains of sleep (quality, latency, duration, efficiency, disturbances, medication use, and daytime dysfunction) and possesses strong psychometric properties. In contrast, while the AIS is simpler and easier to administer, its unidimensional nature, focusing solely on insomnia symptoms, fails to capture information about other types of sleep disorders (18).

Subgroup analysis based on the duration of residence in nursing homes revealed the highest prevalence of sleep disorders (56%) among residents with a length of stay ≥12 months. This elevated risk is not solely attributable to prolonged residence. Contributing factors include: (1) environmental influences within facilities, such as limited activity space and social isolation (19); and (2) age-related factors including declining physiological function, progression or exacerbation of chronic diseases, increased polypharmacy risk (20), and the accumulation of psychological burdens such as loneliness, helplessness, and a sense of loss, all of which collectively heighten the risk of sleep disorders (21, 22).

Consistent with both domestic and international studies (23), our results show that the prevalence of sleep problems among female residents was higher than among males, aligning with reports that females experience sleep problems at 1.3–1.8 times the rate of males. This gender disparity may be linked to the gradual decline or cessation of endogenous estrogen secretion following menopause due to ovarian function decline (24), coupled with women’s heightened sensitivity to inflammatory biomarker responses following sleep deprivation (25).

Temporal trend analysis demonstrated an overall declining trend in the prevalence of sleep disorders among Chinese nursing home residents from 2008 to 2024. This positive shift may be attributed to three key factors: Policy drivers: Implementation of the “Healthy China 2030” blueprint has elevated public awareness of holistic health management. Technological innovation: The proliferation of intelligent sleep monitoring devices (e.g., sleep trackers, polysomnography) and digitalized intervention programs has enabled precise sleep assessment and personalized interventions. Social engagement: Collaborative efforts between healthcare institutions, enterprises, and research organizations in conducting sleep health education campaigns and community intervention projects have fostered a multidimensional support system, collectively promoting improved sleep health nationwide. Given the critical importance of sleep health as a core indicator of older adults well-being (26), we recommend enhancing the sleep health management system within nursing homes by: Optimizing physical infrastructure: Improving acoustic insulation and lighting conditions in living environments. Enhancing service delivery: Establishing dedicated sleep health management teams. Upskilling nursing staff: Providing specialized training in sleep disorder recognition and non-pharmacological interventions (e.g., music therapy, aromatherapy). Implementing personalized care: Developing tailored intervention plans based on individual characteristics. These measures aim to improve sleep quality, promote functional recovery, and ultimately enhance the quality of life for older adults residents.

### Limitations of this study

(1) The included studies exhibited substantial heterogeneity. Although subgroup analyses were conducted based on factors such as gender, geographical region, and duration of nursing home residence, they failed to significantly reduce the heterogeneity. Consequently, the findings of this study may be subject to a certain degree of bias. (2) Among the included literature, the number of studies from some provinces (autonomous regions, municipalities) was limited or even absent, and some subgroups (e.g., Northwestern China, Taiwan/Hong Kong/Macau) contained only a single study. This limits our ability to obtain stable and reliable effect estimates for these specific populations and affects the generalizability of our findings to these groups. Future research targeting these regions is required to validate our findings. (3) All selected studies were cross-sectional in design, which may introduce biases related to implementation and measurement. Furthermore, the results of this review primarily reflect the sleep status of nursing home residents with basic cognitive and communication abilities. They may not be fully representative of the entire nursing home population, particularly those with moderate to severe dementia, a group in which sleep problems are especially prominent. Future studies should adopt more rigorous designs, incorporate standardized cognitive assessments as a basis for inclusion and stratification, and combine caregiver proxy-reported questionnaires with objective sleep monitoring technologies (e.g., actigraphy) to provide a more comprehensive and accurate understanding of sleep issues among nursing home residents across different cognitive statuses.

## Conclusion

The detection rate of sleep disorders among older adults residents in Chinese nursing homes is relatively high (43%), with marked disparities observed across different demographic and geographic groups. Significant attention should be directed toward the sleep health of this vulnerable population. Comprehensive preventive and intervention measures, tailored to the specific characteristics of different subpopulations, must be developed and implemented to effectively improve sleep quality and reduce the burden of sleep disorders.

## Data Availability

Publicly available datasets were analyzed in this study. This data can be found here: All data analyzed during this study are included in this article, and further inquiries can be directed to the corresponding author.

## References

[1] Shi BG Cao Y Wuren TY. Spatial distribution of nursing homes in China and its influencing factors. J South Central Minzu Univ (Hum Soc Sci). (2025) 45:136–148+186-187. doi: 10.19898/j.cnki.42-1704/C.20250424.04

[2] Yan YP Li YL. Challenges and strategies in the transition from healthy aging to active aging in China. Dongyue Tribune. (2022) 43:165–75. 192

[3] Huang D. Healthy China initiative (2019–2030) [Eb/Ol]. Available online at: https://www.gov.cn/xinwen/2019-07/15/content_5409694.htm. (Accessed June 02, 2025)

[4] Riemann D Espie CA Altena E Arnardottir ES Baglioni C Bassetti CLA . The European insomnia guideline: an update on the diagnosis and treatment of insomnia 2023. J Sleep Res. (2023) 32:e14035. doi: 10.1111/jsr.14035, 38016484

[5] World Sleep Day | China Sleep Research Report (2025) [Eb/Ol]. Available online at: https://www.ssap.com.cn/xxydj/detail/34562. (Accessed June 02, 2025).

[6] Wang ZJ Zhao M Chen TW Guo ZL. Prevalence of sleep disorders among the elderly in China: a meta-analysis. Chin Gen Pract. (2022) 25:2036–43.

[7] Pangaribuan SM Wu T-Y Herlianita R Jao YL Lee HC Hasan F . Global occurrence rates of sleep disturbances among institutionalized older adults: a systematic review and meta-analysis. Sleep Med Rev. (2025) 81:102091. doi: 10.1016/j.smrv.2025.102091, 40239318

[8] Hu XW Chen FY Yang RN Fu PB Yuan P. Association between sleep duration and successful aging among middle-aged and older adults in China. Mod Prev Med. (2025) 52:1251–6. Available at: http://xdyfyxzz.paperopen.com/

[9] Chen L Zhang QY Lu YY Peng C Cai BX . Association between sleep duration and falls among middle-aged and older adults in China. Mod Prev Med. (2025) 52:1300–5. Available at: http://xdyfyxzz.paperopen.com/

[10] Lin LX Zeng QC Guo YY Liang RX Wu H Shao YP. Impact of sleep duration patterns on activities of daily living among middle-aged and older adults. Chin J Rehabil Theory Pract. (2025) 31:331–8. Available at: www.cjrtponline.com

[11] Zeng XT Liu H Chen X Leng WD. Meta-analysis series (IV): quality assessment tools for observational studies. Chinese J Evid Bases Cardiovasc Med. (2012) 4:297–9.

[12] Stang A. Critical evaluation of the Newcastle-Ottawa scale for the assessment of the quality of nonrandomized studies in meta-analyses. Eur J Epidemiol. (2010) 25:603–5. doi: 10.1007/s10654-010-9491-z, 20652370

[13] Ye S Chen YH Xie QB. Meta-analysis and software implementation for single incidence rates of 0 or 1. Chin J Health Stat. (2022) 39:316–20.

[14] Kim DE Yoon JY. Factors that influence sleep among residents in long-term care facilities. Int J Environ Res Public Health. (2020) 17:1889. doi: 10.3390/ijerph17061889, 32183274 PMC7142890

[15] Li J Yang B Varrasse M Li K. Sleep among long-term care residents in China: a narrative review of literature. Clin Nurs Res. (2018) 27:35–60. doi: 10.1177/1054773816673175, 27729401 PMC5996244

[16] Kent de Grey RG Uchino BN Trettevik R Cronan S Hogan JN. Social support and sleep: a meta-analysis. Health Psychol. (2018) 37:787–98. doi: 10.1037/hea0000628, 29809022

[17] Pang M Wang J Zhao M Chen R Liu H Xu X . The migrant-local difference in the relationship between social support, sleep disturbance, and loneliness among older adults in China: cross-sectional study. JMIR Public Health Surveill. (2024) 10:e49253. doi: 10.2196/49253, 38194253 PMC10806446

[18] Ma L Shi J. Potential indicators for diagnosing insomnia: interpretation of the Wfsbp consensus statement. Chin J Evid Based Med. (2024) 24:845–52. Available at: https://cluster.hxyx.com/

[19] Štefan L Vrgoč G Rupčić T Sporiš G Sekulić D. Sleep duration and sleep quality are associated with physical activity in elderly people living in nursing homes. Int J Environ Res Public Health. (2018) 15:2512. doi: 10.3390/ijerph15112512, 30423981 PMC6266288

[20] Zhao YL Zhang YX Hou LS Xia X Dong BR. Correlation between potentially inappropriate medication and physical functional decline among elderly in nursing homes in Chengdu. Chin J Gerontol. (2023) 43:2262–7.

[21] Peng SS Liu JY Luo RZ Dai LY Hu JX Liu YH. Research progress on sense of belonging among elderly in nursing homes. Chin J Nurs. (2024) 59:1201–4.

[22] Huang P Wang S Hu SH Huang PH Wang SY Chuang YH. Older residents’ perceptions of loneliness in long-term care facilities: a qualitative study. Int J Ment Health Nurs. (2022) 31:601–10. doi: 10.1111/inm.12979, 35118782

[23] Wang P Song L Wang K Han X Cong L Wang Y . Prevalence and associated factors of poor sleep quality among Chinese older adults living in a rural area: a population-based study [J/Ol]. Aging Clin Exp Res. (2020) 32:125–31. doi: 10.1007/s40520-019-01171-0, 30919262 PMC6974488

[24] Chen GH Deng LY Du YJ Huang ZL Jiang F Jin FR . Expert consensus on diagnosis and treatment of insomnia in specific populations. Zhongguo Linchuang Yaolixue Yu Zhiliao Zazhi. (2024) 29:841–52.

[25] Lian Y Zhang J Jia C-X. Sleep duration change and cognitive function: a national cohort study of Chinese people older than 45 years. J Nerv Ment Dis. (2020) 208:498–504. doi: 10.1097/NMD.0000000000001159, 32187126

[26] Liu Y Ji J Du W Cao Q Su C He Y . Healthy sleep without insomnia may go beyond sleep duration for achieving successful aging in Chinese older adults: a cross-sectional study. BMC Geriatr. (2025) 25:386. doi: 10.1186/s12877-025-06044-y, 40442604 PMC12121161

[27] Hao CM Wang L Xu YX Li X Li ZS. Analysis of influencing factors of sleep disorders among elderly in nursing homes in Beijing. Beijing Med J. (2023) 45:929–33. Available at: www.bjyxh.org.cn

[28] Han YM Wang Z Sun HR Sun ZB Yan JJ Wang XY. Influencing factors of sleep quality in elderly with different traditional Chinese medicine constitutions. Chin J Gerontol. (2023) 43:6119–21.

[29] Zhu FY Li CH Song FB Chen WX Wang DH. Investigation and analysis of sleep quality and influencing factors among elderly in a nursing home in Hangzhou. Health Res. (2022) 42:383–386+390.

[30] Wu WW Jiang XY Zhang X Zhu XL. Sleep quality and influencing factors among elderly in nursing homes in Fuzhou. Chin J Nurs. (2016) 51:352–5.

[31] Gong LC Chen CX Yao XJ Yin YN Han WT. Correlation analysis between sleep disorders and depression among elderly in apartments for the aged. Mod Prev Med. (2012) 39:4175–8. Available at: http://xdyfyxzz.paperopen.com/

[32] Zhu WF. Correlation between sleep quality and social support among elderly in apartments for the aged. Chin J Health Care Med. (2015) 17:315–6.

[33] Huang DZ Ding YB Wang Y She Q Liu H. Investigation on sleep quality and general well-being of elderly in nursing homes in Gaoqiao community, Shanghai. Med Soc. (2019) 32:125–8.

[34] Yu CF Quan HS. Investigation on current sleep quality and related factors among elderly in nursing homes in Siping. Sci Technol West China. (2011) 10:32.

[35] Liu XY Li L Wang Y Wen N. Investigation on sleep quality and influencing factors among elderly in nursing homes in Weifang. Chin J Geriatr Care. (2023) 21:90–3.

[36] Wang YJ Gui Q Zhang Q Chen Y Li WT Ding H . Functional status and influencing factors of elderly in nursing homes. Chin Gen Pract. (2019) 22:468–72.

[37] Yang ZH Zhao M Yang Y Li M Wang KF. Sleep trajectories and their predictors among elderly in nursing homes. Chin Nurs Manage. (2021) 21:503–8.

[38] Wu F Wang YQ Gao J Huang LQ Li M Wang KF. Prediction of changes in activities of daily living trajectories by sleep quality among elderly in nursing homes. J Nurs Sci. (2020) 35:66–9. Available at: www.hlxzz.com.cn

[39] Wang XY Min B Zheng P. Correlation between sleep quality and depression among elderly in nursing homes. Chin J Gerontol. (2024) 44:3303–7.

[40] Cai ZZ Wang XX Luo YT Lin R Li H. Sleep quality and influencing factors among elderly with mild cognitive impairment in nursing homes. J Nurs Sci. (2022) 37:79–81. Available at: www.hlxzz.com.cn

[41] Li YK Wang ZW Yin X. Sleep quality and its relationship with depressive mood among elderly in nursing homes. J Nurs Manag. (2012) 12:697–9.

[42] Gao LN Liu YX Liu J Xin TT. Impact of depression levels on the relationship between sleep quality and cognitive function among elderly in nursing homes. Chin J Health Educ. (2023) 39:790–5.

[43] Zhao QQ Zhang L Yu L Xia QH Jiang Y. Survey on sleep quality of elderly in nursing homes in Changning District. Prev Med. (2025) 37:408–12. Available at: http://www.zjyfyxzz.com/

[44] Xia JH Liu WC Jiang JP. Current status and influencing factors of sleep disorders among elderly in nursing homes in Wenzhou. Chin J Med Doctors. (2018) 20:466–8.

[45] Chen QX Zhang Q Zhao Y Dong LN. Investigation on sleep quality and influencing factors among elderly in nursing homes. Qilu Journal of Nursing. (2014) 20:6–9.

[46] Wu JJ Lin Q Rong X Fu MX Zhang XM Liu LF . Correlation among frailty, medication adherence, and sleep quality in elderly hypertensive patients in nursing homes in Sichuan Province. Med Soc. (2021) 34:56–60.

[47] Nie Y Hu Z Zhu T Xu H. A cross-sectional study of the prevalence of and risk factors for suicidal ideation among the elderly in nursing homes in Hunan Province, China. Front Psych. (2020) 11:339. doi: 10.3389/fpsyt.2020.00339, 32477170 PMC7241427

[48] Zhang J Zhang Y Luan Z Zhang X Jiang H Wang A. A study on depression of the elderly with different sleep quality in pension institutions in northeastern China. BMC Geriatr. (2020) 20:374. doi: 10.1186/s12877-020-01777-4, 32993532 PMC7526187

[49] Gao L Yang J Liu J Xin T Liu Y. Activities of daily living and depression in Chinese elderly of nursing homes: a mediation analysis. Psychol Res Behav Manag. (2023) 16:29–38. doi: 10.2147/PRBM.S394787, 36636291 PMC9831252

[50] Wang Q Zan C Jiang F Shimpuku Y Chen S. Association between loneliness and its components and cognitive function among older Chinese adults living in nursing homes: a mediation of depressive symptoms, anxiety symptoms, and sleep disturbances. BMC Geriatr. (2022) 22:959. doi: 10.1186/s12877-022-03661-9, 36514018 PMC9746079

[51] Liu S Hu Z Guo Y Zhou F Li S Xu H. Association of sleep quality and nap duration with cognitive frailty among older adults living in nursing homes. Front Public Health. (2022) 10:963105. doi: 10.3389/fpubh.2022.963105, 36091504 PMC9453392

[52] Hu Q Song Y Wang S Lin L Ke Y Zhang P. Association of subjective cognitive complaints with poor sleep quality: a cross-sectional study among Chinese elderly. Int J Geriatr Psychiatry. (2023) 38:e5956. doi: 10.1002/gps.5956, 37329227

[53] Liu S Chow IHI Lu L Ren YM Yang HL Jian SY . Comparison of sleep disturbances between older nursing home residents in high- and low-altitude areas. J Geriatr Psychiatry Neurol. (2020) 33:370–6. doi: 10.1177/0891988719892335, 31838930

[54] Jiang Y Xia Q Wang J Zhou P Jiang S Diwan VK . Insomnia, benzodiazepine use, and falls among residents in long-term care facilities. Int J Environ Res Public Health. (2019) 16:4623. doi: 10.3390/ijerph16234623, 31766368 PMC6926709

[55] Sun WJ Liu YJ. The impact of social support on sleep quality in elderly care institutions in Northeast China: the chain-mediating effect of psychological adjustment and coping style. Patient Prefer Adherence. (2024) 18:1119–30. doi: 10.2147/PPA.S461449, 38863944 PMC11164688

[56] Wang H Wang J Xie B Liu B. Multi-dimensional frailty and its risk factors among older residents in long-term care facilities in Shanghai, China. Int J Nurs Sci. (2021) 8:298–303. doi: 10.1016/j.ijnss.2021.06.003, 34307778 PMC8283709

[57] Tsai Y Wong TK Ku Y. Self-care management of sleep disturbances and risk factors for poor sleep among older residents of Taiwanese nursing homes. J Clin Nurs. (2008) 17:1219–26. doi: 10.1111/j.1365-2702.2007.02020.x, 18266847

[58] Wen B Li Y Zhang M Xu H. Association of dysphagia and loneliness and their interaction with sleep quality among older adults in nursing homes: a cross-sectional study. PLoS One. (2024) 19:e0311024. doi: 10.1371/journal.pone.0311024, 39325814 PMC11426441

[59] Hou T. Depressive symptoms, sleep quality, and pain are associated with frailty in nursing home residents during the Covid-19 pandemic. Pain Manag Nurs. (2024) 25:241–8. doi: 10.1016/j.pmn.2024.02.001, 38413256

[60] Zhu W Wang Y Tang J Wang F. Sleep quality as a mediator between family function and life satisfaction among Chinese older adults in nursing home. BMC Geriatr. (2024) 24:379. doi: 10.1186/s12877-024-04996-1, 38684958 PMC11059730

[61] Mou H Xu D Zhu S Zhao M Wang Y Wang K. The sleep patterns and their associations with mental health among nursing home residents: a latent profile approach. BMC Geriatr. (2023) 23:468. doi: 10.1186/s12877-023-04124-5, 37537539 PMC10401828

