# Supplementary material for: Prevalence of sleep disorders among older adults in Chinese older adults care institutions: a systematic review and meta-analysis
Source: Front Public Health. 2025 Dec 3;13:1664136. doi: 10.3389/fpubh.2025.1664136 (PMC12708321; doi:10.3389/fpubh.2025.1664136)

**Effects of non-pharmacological interventions on sleep in chronic low back pain: A systematic review and meta-analysis of randomised controlled trials**

**Supplementary materials**

[Appendix 1: PRISMA 2020 checklist 3](#_Toc126917259)

[Appendix 2: Search terms and database search results (from database inception to 2 June 2021) 7](#_Toc126917260)

[Appendix 3: GRADE criteria 9](#_Toc126917261)

[Appendix 4: Details of interventions, outcome measures not included in meta-analyses, sources of funding, and conflicts of interest 10](#_Toc126917262)

[Appendix 5: Data handing 18](#_Toc126917263)

[Appendix 6: Summary of amendments to information provided at registration or in the protocol. 20](#_Toc126917264)

[Appendix 7: Measurement instruments used in included studies 21](#_Toc126917265)

[Appendix 8: Risk of Bias Assessment 24](#_Toc126917266)

[sFig. 8.1. Risk of bias assessment for included studies (by the number of studies) 24](#_Toc126917267)

[sFig. 8.2. Risk of bias assessment for included studies (by number of participants) 24](#_Toc126917268)

[Appendix 9: Sensitivity analysis and funnel plot for sleep outcomes 25](#_Toc126917269)

[sFig. 9.1. Outlier analysis for sleep 25](#_Toc126917270)

[sFig. 9.2. Influential study analysis (leave-one-out) for sleep 25](#_Toc126917271)

[sFig. 9.3. Funnel plot for sleep 26](#_Toc126917272)

[sFig. 9.4. Sensitivity analysis –pairwise analysis for sleep with crossover RCTs excluded 26](#_Toc126917273)

[sFig. 9.5. Influential study analysis (leave-one-out) for sleep questionnaires 27](#_Toc126917274)

[sFig. 9.6. Outlier analysis for sleep questionnaires (Category of sleep questionnaires – Composite sleep measures) 28](#_Toc126917275)

[Appendix 10: Sensitivity analysis and funnel plot for pain intensity 29](#_Toc126917276)

[sFig. 10.1. Outlier analysis for pain intensity 29](#_Toc126917277)

[sFig. 10.2. Influential study analysis (leave-one-out) for pain intensity 29](#_Toc126917278)

[sFig. 10.3. Sensitivity analysis –pairwise analysis for pain intensity with crossover RCTs excluded 30](#_Toc126917279)

[sFig. 10.4. Funnel plot for pain intensity 30](#_Toc126917280)

[Appendix 11: Sensitivity analysis for back-related disability 31](#_Toc126917281)

[sFig. 11.1. Outlier analysis for back-related disability 31](#_Toc126917282)

[sFig. 11.2. Influential study analysis (leave-one-out) for back-related disability 31](#_Toc126917283)

[sFig. 11.3. Funnel plot for back-related disability 32](#_Toc126917284)

[Appendix 12: Subgroup analysis (non-specific chronic low back pain) 33](#_Toc126917285)

[sFig. 12.1. Effect of non-pharmacological interventions on sleep in non-specific chronic low back pain 33](#_Toc126917286)

[sFig. 12.2. Effect of non-pharmacological interventions on pain intensity in non-specific chronic low back pain 33](#_Toc126917287)

[sFig. 12.3. Effect of non-pharmacological interventions on back-related disability in non-specific chronic low back pain 34](#_Toc126917288)

[Appendix 13: Subgroup analysis (sham treatment) 35](#_Toc126917289)

[sFig. 13.1. Effect of non-pharmacological interventions vs. sham treatment on sleep 35](#_Toc126917290)

[sFig. 13.2. Effect of non-pharmacological interventions vs. sham treatment on pain intensity 35](#_Toc126917291)

Appendix 1: PRISMA 2020 checklist

(template downloaded from <http://prisma-statement.org/PRISMAStatement/Checklist.aspx>)

| **Section and Topic** | **Item #** | **Checklist item** | **Location where item is reported** |
| --- | --- | --- | --- |
| **TITLE** | | |  |
| Title | 1 | Identify the report as a systematic review. |  |
| **ABSTRACT** | | |  |
| Abstract | 2 | See the PRISMA 2020 for Abstracts checklist. |  |
| **INTRODUCTION** | | |  |
| Rationale | 3 | Describe the rationale for the review in the context of existing knowledge. |  |
| Objectives | 4 | Provide an explicit statement of the objective(s) or question(s) the review addresses. |  |
| **METHODS** | | |  |
| Eligibility criteria | 5 | Specify the inclusion and exclusion criteria for the review and how studies were grouped for the syntheses. | Method: eligibility criteria; synthesis methods |
| Information sources | 6 | Specify all databases, registers, websites, organisations, reference lists and other sources searched or consulted to identify studies. Specify the date when each source was last searched or consulted. | Method: search strategy |
| Search strategy | 7 | Present the full search strategies for all databases, registers and websites, including any filters and limits used. | Method: search strategy; Appendix 2 |
| Selection process | 8 | Specify the methods used to decide whether a study met the inclusion criteria of the review, including how many reviewers screened each record and each report retrieved, whether they worked independently, and if applicable, details of automation tools used in the process. | Method: study selection and data extraction |
| Data collection process | 9 | Specify the methods used to collect data from reports, including how many reviewers collected data from each report, whether they worked independently, any processes for obtaining or confirming data from study investigators, and if applicable, details of automation tools used in the process. | Method: study selection and data extraction |
| Data items | 10a | List and define all outcomes for which data were sought. Specify whether all results that were compatible with each outcome domain in each study were sought (e.g. for all measures, time points, analyses), and if not, the methods used to decide which results to collect. | Method: eligibility criteria; study selection and data extraction |
|  | 10b | List and define all other variables for which data were sought (e.g. participant and intervention characteristics, funding sources). Describe any assumptions made about any missing or unclear information. | Method: study selection and data extraction; synthesis methods |
| Study risk of bias assessment | 11 | Specify the methods used to assess risk of bias in the included studies, including details of the tool(s) used, how many reviewers assessed each study and whether they worked independently, and if applicable, details of automation tools used in the process. | Method: risk of bias and certainty of evidence assessment |
| Effect measures | 12 | Specify for each outcome the effect measure(s) (e.g. risk ratio, mean difference) used in the synthesis or presentation of results. | Method: synthesis methods |
| Synthesis methods | 13a | Describe the processes used to decide which studies were eligible for each synthesis (e.g. tabulating the study intervention characteristics and comparing against the planned groups for each synthesis (item #5)). | Method: synthesis methods |
|  | 13b | Describe any methods required to prepare the data for presentation or synthesis, such as handling of missing summary statistics, or data conversions. | Method: synthesis methods |
|  | 13c | Describe any methods used to tabulate or visually display results of individual studies and syntheses. | Method: synthesis methods |
|  | 13d | Describe any methods used to synthesize results and provide a rationale for the choice(s). If meta-analysis was performed, describe the model(s), method(s) to identify the presence and extent of statistical heterogeneity, and software package(s) used. | Method: synthesis methods |
|  | 13e | Describe any methods used to explore possible causes of heterogeneity among study results (e.g. subgroup analysis, meta-regression). | Method: assessment of heterogeneity and publication bias |
|  | 13f | Describe any sensitivity analyses conducted to assess robustness of the synthesized results. | Method: sensitivity analysis |
| Reporting bias assessment | 14 | Describe any methods used to assess risk of bias due to missing results in a synthesis (arising from reporting biases). | Method: assessment of heterogeneity and publication bias |
| Certainty assessment | 15 | Describe any methods used to assess certainty (or confidence) in the body of evidence for an outcome. | Method: risk of bias and certainty of evidence assessment |
| **RESULTS** | | |  |
| Study selection | 16a | Describe the results of the search and selection process, from the number of records identified in the search to the number of studies included in the review, ideally using a flow diagram. | Results: search results; Fig. 1 |
|  | 16b | Cite studies that might appear to meet the inclusion criteria, but which were excluded, and explain why they were excluded. | - |
| Study characteristics | 17 | Cite each included study and present its characteristics. | Results: search results; characteristics of included studies |
| Risk of bias in studies | 18 | Present assessments of risk of bias for each included study. | Fig. 2; Supplementary Appendix 8 |
| Results of individual studies | 19 | For all outcomes, present, for each study: (a) summary statistics for each group (where appropriate) and (b) an effect estimate and its precision (e.g. confidence/credible interval), ideally using structured tables or plots. | Fig. 3; Fig. 4; Fig. 5; Fig 6; Table 2 |
| Results of syntheses | 20a | For each synthesis, briefly summarise the characteristics and risk of bias among contributing studies. | Results: Table 2; primary outcomes; secondary outcomes |
|  | 20b | Present results of all statistical syntheses conducted. If meta-analysis was done, present for each the summary estimate and its precision (e.g. confidence/credible interval) and measures of statistical heterogeneity. If comparing groups, describe the direction of the effect. | Results: Table 2; primary outcomes; secondary outcomes |
|  | 20c | Present results of all investigations of possible causes of heterogeneity among study results. | Results: Table 2; primary outcomes; secondary outcomes; Supplementary Appendix 9-13 |
|  | 20d | Present results of all sensitivity analyses conducted to assess the robustness of the synthesized results. | Results: Table 2; primary outcomes; secondary outcomes; Supplementary Appendix 9-11 |
| Reporting biases | 21 | Present assessments of risk of bias due to missing results (arising from reporting biases) for each synthesis assessed. | Fig. 2; Supplementary sFig. 9.3; sFig. 10.4; sFig. 11.3 |
| Certainty of evidence | 22 | Present assessments of certainty (or confidence) in the body of evidence for each outcome assessed. | Table 2 |
| **DISCUSSION** | | |  |
| Discussion | 23a | Provide a general interpretation of the results in the context of other evidence. | Discussion |
|  | 23b | Discuss any limitations of the evidence included in the review. | Discussion: strengths and limitations |
|  | 23c | Discuss any limitations of the review processes used. | Discussion: strengths and limitations |
|  | 23d | Discuss implications of the results for practice, policy, and future research. | Discussion: clinical implications; strengths and limitations |
| **OTHER INFORMATION** | | |  |
| Registration and protocol | 24a | Provide registration information for the review, including register name and registration number, or state that the review was not registered. | Abstract, Methods |
|  | 24b | Indicate where the review protocol can be accessed, or state that a protocol was not prepared. | Brief protocol via PROSPERO registration |
|  | 24c | Describe and explain any amendments to information provided at registration or in the protocol. | Supplementary Appendix 6 |
| Support | 25 | Describe sources of financial or non-financial support for the review, and the role of the funders or sponsors in the review. | Declaration |
| Competing interests | 26 | Declare any competing interests of review authors. | Declaration |
| Availability of data, code and other materials | 27 | Report which of the following are publicly available and where they can be found: template data collection forms; data extracted from included studies; data used for all analyses; analytic code; any other materials used in the review. | Data used for all analyses and analytic codes were included in the Supplementary File. |

Appendix 2: Search terms and database search results (from database inception to 2 June 2021)

| **PubMed** | | |
| --- | --- | --- |
| **Search** | **Query** | **Hits** |
| #1 | "back pain"[MeSH Terms] OR "low back pain"[MeSH Terms] OR "back pain* "[Title/Abstract] OR "lumbago"[Title/Abstract] OR "backache*"[Title/Abstract] OR "back ache*"[Title/Abstract] OR "spinal stenosis"[Title/Abstract] OR "canal stenosis"[Title/Abstract] OR "lumbar stenosis"[Title/Abstract] OR "lateral stenosis "[Title/Abstract] OR "neurogenic claudication"[Title/Abstract] OR "radiculopathy"[Title/Abstract] OR "radicular pain"[Title/Abstract] OR "spondylolisthesis"[Title/Abstract] OR "spondylosis"[Title/Abstract] OR "sciatica"[Title/Abstract] OR "intervertebral disc displacement"[Title/Abstract] OR "spinal nerve roots"[Title/Abstract] OR "neurologic signs "[Title/Abstract] OR "paresthesia"[Title/Abstract] OR "paraesthesia"[Title/Abstract] OR "numbness"[Title/Abstract] | 106522 |
| #2 | "sleep"[MeSH Terms] OR "sleep initiation and maintenance disorders"[MeSH Terms] OR "sleep*" OR "insomnia" OR "nap" OR "naps" OR "napping" | 265333 |
| #3 | ("randomized controlled trial"[Publication Type] OR "controlled clinical trial"[Publication Type] OR "randomized"[Title/Abstract] OR "placebo"[Title/Abstract] OR "randomly"[Title/Abstract] OR "trial"[Title] OR "clinical trials as topic"[MeSH Terms:noexp]) NOT (animals[MeSH Terms] NOT humans[MeSH Terms]) | 1272008 |
| #4 | animals[MeSH Terms] NOT humans[MeSH Terms] | 4835945 |
| #5 | #1 AND #2 AND #3 NOT #4 | 358 |
| **CINAHL** | | |
| **Search** | **Query** | **Hits** |
| #1 | MH "back pain+" OR MH "low back pain+" OR TI ("back pain*" OR "lumbago" OR "backache*" OR "back ache*" OR "spinal stenosis" OR "canal stenosis" OR "lumbar stenosis" OR "lateral stenosis " OR "neurogenic claudication" OR "radiculopathy" OR "radicular pain" OR "spondylolisthesis" OR "spondylosis" OR "sciatica" OR "intervertebral disc displacement" OR "spinal nerve roots" OR "neurologic signs " OR "paresthesia" OR "paraesthesia" OR "numbness") OR AB ("back pain*" OR "lumbago" OR "backache*" OR "back ache*" OR "spinal stenosis" OR "canal stenosis" OR "lumbar stenosis" OR "lateral stenosis " OR "neurogenic claudication" OR "radiculopathy" OR "radicular pain" OR "spondylolisthesis" OR "spondylosis" OR "sciatica" OR "intervertebral disc displacement" OR "spinal nerve roots" OR "neurologic signs " OR "paresthesia" OR "paraesthesia" OR "numbness") | 51148 |
| #2 | MH "sleep+" OR MH "sleep initiation and maintenance disorders+" OR "sleep*" OR "insomnia" OR "nap" OR "naps" OR "napping" | 87266 |
| #3 | PT("randomized controlled trial" OR "controlled clinical trial") OR TI("randomized" OR "placebo" OR "randomly" OR "trial") OR AB("randomized" OR "placebo" OR "randomly" | 371753 |
| #4 | MH "animals+" NOT MH "human+" | 87756 |
| #5 | #1 AND #2 AND #3 NOT #4 | 148 |
| **SPORTDiscus** | | |
| **Search** | **Query** | **Hits** |
| #1 | DE "backache+" OR TI ("back pain*" OR "lumbago" OR "backache*" OR "back ache*" OR "spinal stenosis" OR "canal stenosis" OR "lumbar stenosis" OR "lateral stenosis " OR "neurogenic claudication" OR "radiculopathy" OR "radicular pain" OR "spondylolisthesis" OR "spondylosis" OR "sciatica" OR "intervertebral disc displacement" OR "spinal nerve roots" OR "neurologic signs " OR "paresthesia" OR "paraesthesia" OR "numbness") OR AB ("back pain*" OR "lumbago" OR "backache*" OR "back ache*" OR "spinal stenosis" OR "canal stenosis" OR "lumbar stenosis" OR "lateral stenosis " OR "neurogenic claudication" OR "radiculopathy" OR "radicular pain" OR "spondylolisthesis" OR "spondylosis" OR "sciatica" OR "intervertebral disc displacement" OR "spinal nerve roots" OR "neurologic signs " OR "paresthesia" OR "paraesthesia" OR "numbness") | 11290 |
| #2 | DE "sleep+" OR "sleep*" OR "insomnia" OR "nap" OR "naps" OR "napping" | 14747 |
| #3 | TI("randomized" OR "placebo" OR "randomly" OR "trial") OR AB("randomized" OR "placebo" OR "randomly") | 54327 |
| #4 | #1 AND #2 AND #3 | 30 |
| **PsycINFO** | | |
| #1 | MA "back pain+" OR MA "low back pain+" OR TI ("back pain*" OR "lumbago" OR "backache*" OR "back ache*" OR "spinal stenosis" OR "canal stenosis" OR "lumbar stenosis" OR "lateral stenosis " OR "neurogenic claudication" OR "radiculopathy" OR "radicular pain" OR "spondylolisthesis" OR "spondylosis" OR "sciatica" OR "intervertebral disc displacement" OR "spinal nerve roots" OR "neurologic signs " OR "paresthesia" OR "paraesthesia" OR "numbness") OR AB ("back pain*" OR "lumbago" OR "backache*" OR "back ache*" OR "spinal stenosis" OR "canal stenosis" OR "lumbar stenosis" OR "lateral stenosis " OR "neurogenic claudication" OR "radiculopathy" OR "radicular pain" OR "spondylolisthesis" OR "spondylosis" OR "sciatica" OR "intervertebral disc displacement" OR "spinal nerve roots" OR "neurologic signs " OR "paresthesia" OR "paraesthesia" OR "numbness") | 8007 |
| #2 | MH "sleep+" OR MH "sleep initiation and maintenance disorders+" OR "sleep*" OR "insomnia" OR "nap" OR "naps" OR "napping" | 81537 |
| #3 | TI("randomized" OR "placebo" OR "randomly" OR "trial") OR AB("randomized" OR "placebo" OR "randomly") | 179418 |
| #4 | MA "animals+" NOT MA "human+" | 42284 |
| #5 | #1 AND #2 AND #3 NOT #4 | 56 |
| **EMBASE** | | |
| **Search** | **Query** | **Hits** |
| #1 | 'back pain'/exp OR 'low back pain'/exp OR 'back pain*':ab,ti OR 'lumbago':ab,ti OR 'backache*':ab,ti OR 'back ache*':ab,ti OR 'spinal stenosis':ab,ti OR 'canal stenosis':ab,ti OR 'lumbar stenosis':ab,ti OR 'lateral stenosis':ab,ti OR 'neurogenic claudication':ab,ti OR 'radiculopathy':ab,ti OR 'radicular pain':ab,ti OR 'spondylolisthesis':ab,ti OR 'spondylosis':ab,ti OR 'sciatica':ab,ti OR 'intervertebral disc displacement':ab,ti OR 'spinal nerve roots':ab,ti OR 'neurologic signs':ab,ti OR 'paresthesia':ab,ti OR 'paraesthesia':ab,ti OR 'numbness':ab,ti | 184874 |
| #2 | 'sleep'/exp OR 'sleep*' OR 'insomnia' OR 'nap' OR 'naps' OR 'napping' | 492802 |
| #3 | 'randomized controlled trial':it OR 'controlled clinical trial':it OR 'randomized':ab,ti OR 'placebo':ab,ti OR 'randomly':ab,ti OR 'trial':ti OR 'clinical trials'/de | 1562710 |
| #4 | 'animal'/exp NOT 'human'/exp | 5616914 |
| #5 | #1 AND #2 AND #3 NOT #4 | 2836 |
| **CENTRAL** | | |
| **Search** | **Query** | **Hits** |
| #1 | (MeSH descriptor: [Low Back Pain] explode all trees) OR (MeSH descriptor: [Back Pain] explode all trees) OR ("back pain*" OR "lumbago" OR "backache*" OR "back ache*" OR "spinal stenosis" OR "canal stenosis" OR "lumbar stenosis" OR "lateral stenosis " OR "neurogenic claudication" OR "radiculopathy" OR "radicular pain" OR "spondylolisthesis" OR "spondylosis" OR "sciatica" OR "intervertebral disc displacement" OR "spinal nerve roots" OR "neurologic signs " OR "paresthesia" OR "paraesthesia" OR "numbness"):ti,ab,kw | 24838 |
| #2 | (MeSH descriptor: [Sleep] explode all trees) OR (MeSH descriptor: [Sleep Initiation and Maintenance Disorders] explode all trees) OR ("sleep*" OR "insomnia" OR "nap" OR "naps" OR "napping") | 47663 |
| #3 | (MeSH descriptor: [Animals] explode all trees) NOT (MeSH descriptor: [Humans] explode all trees) | 59 |
| #4 | #1 AND #2 NOT #3 | 1504 |
| #5 | #4 AND Limit: Trials | 1462 |

****Appendix 3****: ****GRADE criteria****

| **GRADE Domain** | **Criteria used in this review** |
| --- | --- |
| **Risk of bias** | Downgraded by one level if 50% of studies or 50% of n-randomised had high risk of bias, or 75% of studies or 75% of n-randomised was from studies with some concerns.  Downgraded by two levels if all studies had high risk of bias |
| **Inconsistency** | Downgraded by one level if substantial heterogeneity was present. For example, effect estimates varied widely across studies, 95% CI had showed minimal or no overlap, I^2^>50%, or the prediction interval showing treatment effects favouring both directions. |
| **Indirectness** | Downgraded by one level if any evidence supporting the meta-analysis came from studies not directly related to the PICOS criteria. |
| **Imprecision** | Downgraded by one level if the 95% confidence interval of the effect estimate includes no effect or the sample size <800 in total (<400 per group). |
| **Publication bias** | Downgraded by one level if evidence of asymmetry in the funnel plots, or statistical evidence (p<.05) from the modified Eggers test. |

Appendix 4: Details of interventions, outcome measures not included in meta-analyses, sources of funding, and conflicts of interest

| **Author, year** | **Experimental Treatment details** | **Control group** | **Adverse events** | **Post-intervention outcome measures not included in meta-analyses and reasons for exclusion (sleep [S], pain intensity [P], and back-related disability [D])** | **Sources of funding and conflicts of interest (CoI)** |
| --- | --- | --- | --- | --- | --- |
| **Acupuncture** | | | | | |
| Carlsson 2001 [60] | **Acupuncture**^a^  8 × weekly sessions (8 weeks)  **Manual acupuncture**  Needle acupuncture was given to points of the lower back, lower limbs, forearms and/or hands mostly at a depth of 2 to 3 cm. The “de-qi” feeling of numbness, soreness, heaviness, and warmth was sought in all instances.  **Electroacupuncture** Following 2-3 sessions of manual acupuncture, electrical stimulation of four needles was given to points of low back with alternating frequency of 2/15Hz. | Sham transcutaneous electrical nerve stimulation (TENS). Inactive electrodes were placed on the skin over the most intensely painful area in the low back. | n*=*0 | **[S] Sleep diary**  Sleep disturbance in the acupuncture group was significantly decreased (p=.001); while sleep disturbance in the control group remained unchanged (p=.317).  *Reason*: Post-intervention mean and SD were not available and could not be computed based on published data. Study author advised ordinal data were analysed and the original data could not be retrieved as they have been disposed 20 years post-trial accordingly to the applicable guideline.  **[P] VAS-100**  A significant decrease in mean weekly pain intensities (combined morning and evening values) measured at 1-month post-intervention in the acupuncture groups (mean [SD]: 50.63 [23.75]) compared with the placebo group (61.76 [25.07]).  *Reason*: As sleep outcomes were not include in the primary meta-analysis, pain intensity outcomes were also omitted from secondary meta-analysis. | NR |
| **Education** | | | | | |
| Williams 2018 [75] | **Heathy lifestyle intervention**  26-week follow-up after randomisation  The intervention involved (1) brief telephone advice on LBP, weight loss, and physical activity for reducing LBP, (2) offer of a face-to-face 60-min clinical consultation including LBP education and behaviour change techniques, and (3) referral to a 6-month telephone-based healthy lifestyle coaching service (10 individually tailored coaching calls). | Usual care | n*=*32^b^ | **[S] PSQI (subjective sleep quality item only)**  Exp: n=5/79 had poor sleep  Con: n=8/80 had poor sleep  RR [95% CI] = 0.50 [-0.67, 1.66]^c^  *Reason*: A meta-analysis was not performed due to insufficient number of studies with RR (<5) and mean and SD could not be calculated from published data.  **[P] NRS-10**  There was no difference between the treatment group (mean [SD]: 5.8 [2.7]) and control (6.3 [2.4]) for pain intensity over 6 months (mean difference [95%CI]: 0.3 [-0.4, 1.0], p=.36).  **[D] RMDQ**  There was no difference between the treatment group (mean [SD]: 13.9 [6.5]) and control (14.7 [5.9]) for pain intensity over 6 months (mean difference [95%CI]: -0.1 [-1.7, 1.5], p≥.05).  *Reason*: As sleep outcomes were not include in the primary meta-analysis, pain intensity and back-related disability outcomes were also omitted from secondary meta-analysis. | *Funding*: The University of Newcastle and the Hunter Medical Research Institute. Author declared the sponsors had no involvement in the design and conduct of the study; collection, management, analysis, and interpretation of the data; preparation, review, or approval of the manuscript; and decision to submit the manuscript for publication  *CoI*: Author declared not having CoI. |
| Licciardone 2020 [71] | **eHealth intervention**  3-month follow up after randomisation  Participants received a health-related quality of life report (with a graphic summary and an interpretation guide) that was tailored to each person based on his/her baseline scores on sleep disturbance, pain interference with activities, anxiety, depression, and low energy/fatigue. | Waitlist | NR |  | *Funding*: University of North Texas Health Science Center. Author declared the sponsor had no role in any aspects of study design, data analysis, or submission of the manuscript for publication  *CoI*: Author declared not having CoI. |
| **Electrophysical Therapy** | | | | | |
| Bursali 2021 [59] | **Repetitive transcranial magnetic stimulation (r‑TMS)**  10 × 20-min sessions (5 sessions/week; 2 weeks)  The r-TMS involved non-invasive stimulation of cerebral cortex at 5-Hz (1,000 pulses). | Sham r-TMS (inactive device) | n*=*1 (headache) | **[S] PSQI**  The r-TMS group reported better sleep quality and less sleep disturbance (Mdn [SD] points: 3 [1.7]) compared to the sham r‑TMS group (6 [5.54]), p=.019.  **[P] VAS-10**  The r-TMS group experienced significantly less pain intensity during activity (Mdn [SD] points: 3 [2.06]) compared to the sham r‑TMS group (7.5 [2.72]), p=.016. groups, but not in pain intensity during resting (2.5 [1.99] vs. 5 [2.77]), p=.116 or at night (2 [1.83] vs. 5 [3.46]), p=.260.  **[D] ODI**  The r-TMS group reported less severe back‑related disability (Mdn [SD] points: 21.1 [6.4]) compared to the sham r-TMS group (27.9 [6.9]), p=.035.  *Reason*: Post-intervention mean and SD could not be imputed due to lack of information on the minimum and maximum values. | NR |
| de Teresa 2021 [61] | **Pulsed electromagnetic signals (PEMS) therapy**  10 × 20-min sessions (5 sessions/week; 2 weeks)  Low energy PEMS (840 kHz) was applied to the painful area via non-invasive transcutaneous and dielectric capacitive monopolar transmission. | Sham PEMS (inactive device) | n*=*0 |  | *Funding*: Author declared 'no external funding received'  *CoI*: Author declared not having CoI. |
| Ghoname 1999a [65] | **(1) Percutaneous electrical nerve stimulation (PENS)**  **(2) TENS**  **(3) Flexion-extension exercise**  Crossover RCT with 4 × 3-week treatment blocks (plus 1-week washout period after each treatment block)  9 × 30-min sessions (3 sessions/week)  PENS was administered by insertion of 10 acupuncture-like needles to a depth of 2 to 4 cm in the low back. The probes connected to an electrical generator which produced a unipolar square wave at 4 Hz and a 0.5-millisecond pulse duration. Stimulation was adjusted to maximum tolerable level without muscle contraction.  TENS was administered using four 2.5-cm cutaneous electrodes placed in the dermatomal distribution of pain, with stimulation at 4 Hz and a 0.1-millisecond pulse duration.  Exercise therapy consisted of spinal flexion and extension from sitting performed 30 times during a 30-minute session. | Sham PENS (inactive device) | NR |  | *Funding*: The pilot study was funded by the Forest Park Institute for Pain Management, Texas  *CoI*: Two out of nine authors incorporated a company (PENS Inc) one month after submission of the manuscript to produce PENS unit. |
| Ghoname1999b [66] | **(1) PENS**  **(2) TENS**  Crossover RCT with 3 × 3-week treatment blocks (plus 1-week washout period after each treatment block)  9 × 30-min sessions (3 sessions/week)  PENS was administered by insertion of 10 acupuncture-like needles to a depth of 2 to 4 cm in the low back. The probes connected to an electrical generator which produced a unipolar square wave at 4 Hz and a 0.5-millisecond pulse duration. Stimulation was adjusted to maximum tolerable level without muscle contraction.  TENS was administered using four 2.5-cm cutaneous electrodes placed in the dermatomal distribution of pain, with stimulation at 4 Hz and a 0.1-millisecond pulse duration. | Sham PENS (inactive device) | NR |  | NR |
| Ghoname 1999c [67] | **PENS**  Crossover RCT with 4 × 2-week treatment blocks (plus 1-week washout period after each treatment block)  6 × 30-min sessions (3 sessions/week)  Ten acupuncture needle probes were placed into the soft tissue or muscle to a depth of 2–4 cm in the low back. The probes were connected to an investigational low-output electrical generator. The intensity of the electrical stimulation (100Hz, alternating 15/30Hz, or 4 Hz depending on the assigned group) was adjusted to produce the highest tolerable electrical sensation without muscle contractions (except for the sham treatments). | Sham PENS (inactive device) | NR | **[S] VAS-10 (Sleep quality)**^d^  There was statistically significant difference (p<.05) in percentage improvement in sleep quality at the end of each 2-week treatment block in groups treated with 100Hz (percentage improvement [SD]: 40% [17.98%]), alternating 15/30Hz (60% [24.90%]), or 4 Hz (43% [20.96%]) when compared to the control group (4% [15.87%]).  *Reason*: Post-intervention mean and SD could not be imputed based on available data (only percentage of improvement was reported but no baseline data to enable imputation of post-intervention values).  **[P] VAS-10**  Compared with the sham treatments (mean [SD]: 5.5 [1.8]), the 4-Hz (1.2 [1.2]), 15/30-Hz (1.1 [1.4]), and 100-Hz (1.2 [1.5]) frequencies of PENS therapy also produced statistically greater decreases in the degree of pain at the end of 2-week treatment.  *Reason*: As sleep outcomes were not include in the primary meta-analysis, pain intensity outcomes were also omitted from secondary meta-analysis. | NR |
| Hamza 1999 [69] | **PENS**  Crossover RCT with 4 × 2-week treatment blocks (plus 1 week washout period after each treatment block)  6 × 60-min session (3 sessions/week)  Ten acupuncture needle probes were placed into the soft tissue or muscle to a depth of 2–4 cm in the low back. The probes were connected to an investigational low-output electrical generator and stimulated for a period of 0, 15, 30, or 45 minutes at an alternating frequency of 15 and 30 Hz. | Sham PENS (Inactive device) | NR | **[S] VAS-10 (Sleep quality)**^d^  There was statistically significant difference (p<.05) in percentage improvement in sleep quality at the end of each 2-week treatment block in groups treated for 15, 30, or 45 minutes (25%, 44%, and 40% respectively) when compared to the control group (10%).  **[P] VAS-10**  Compared to sham treatment (0 min; mean [SD]: 5.4 [1.9]), the electrical stimulation for the period of 15, 30, and 45 min produced significant decrease of pain intensity at the end of 2-week treatment (2.0 [1.7], 1.6 [1.8], and 1.5 [1.4] respectively).  *Reason*: Post-intervention mean and SD could not be imputed based on available data (only percentage of improvement was reported but no baseline data to enable imputation of post-intervention values).  *Reason*: As sleep outcomes were not include in the primary meta-analysis, pain intensity outcomes were also omitted from secondary meta-analysis. | NR |
| Omar 2012 [74] | **Pulsed electromagnetic field (PEMF) therapy**  21 × 20-min daily sessions (3 weeks)  The PEMF strengths ranged from 5 to 15 Gauss and the frequency ranged from 7 Hz to 4 kHz. | Sham PEMF (inactive device) | NR | **[S] ODI‑Sleep item**  There was statistically significant difference in sleep disturbance between PEMF and sham PEMF (p=.003).  *Reason*: Post-intervention mean and SD could not be imputed based on available data (only p-value was reported).  **[P] VAS-10**  Compared to sham treatment (mean [SD]: 5.8 [2.7], PEMF significantly decrease pain intensity at the end-of treatment (3.6 [1.5]), p=.024).  *Reason*: As sleep outcomes were not include in the primary meta-analysis, pain intensity outcomes were also omitted from secondary meta-analysis. | *Funding*: NR  *CoI*: Author declared not having CoI. |
| **Exercise (also see Ghoname 1999a) [65]** | | | | | |
| Abadi 2019 [58] | **Aquatic exercise**  24 × 60-min sessions (2 sessions/week; 12 weeks)  The exercise involved walking, jogging, lunging, pedalling, cycling, upper body mobilization and shoulder transvers exercise in shallow water. | No treatment | NR |  | *Funding*: University Research Grant (Sultan Idris Education)  *COI*: Author declared not having CoI. |
| **External Lumbar Support** | | | | | |
| Hagiwara 2017 [68] | **Spinal Underwear**  The experimental group wore the Spinal Underwear (wearable lumbar support) for the first 3 months except bathing and sleeping.  The Spinal Underwear stimulated a tactile sense on the skin of the back while in poor posture aiming to affect the erector spinae muscles and to correct into a better posture. | Waitlist | NR |  | *Funding*: NR  *CoI*: Author declared not having CoI. |
| **Massage (including acupressure)** | | | | | |
| Field 2007 [63] | **Massage**  10 × 30-min sessions (2 sessions/week; 5 weeks)  The massage followed the prescribed techniques and applied to the entire back and legs, and parts of neck and abdomen. | Attention control (Relaxation therapy) | NR |  | *Funding*: Awards and grants from Johnson & Johnson and Biotone to the Touch Research Institutes  *CoI*: NR |
| Hernandez-reif 2001 [70] | **Massage**  10 × 30-min sessions (2 sessions/week; 5 weeks)  The massage followed the prescribed techniques and applied to the entire back and legs, and parts of neck and abdomen. | Attention control (Relaxation therapy) | NR | **[S] Sleep Scale (sleep efficiency and supplementary sleep subscales)**  Higher scores indicate better sleep. Sleep efficiency and supplementary sleep was not statistically different between the massage and the control groups.  *Sleep efficiency*: mean (SD) points: 49.9 (13.6) vs. 38 (18.9).  *Supplementary sleep*: 14.6 (15.6) vs. 17.2 (14.5).  *Reason*: As these two categories were from a reversed scale, the scores could not be pooled with ‘sleep disturbance’ to provide a composite score. Author also could not confirm the maximum possible score for each subscale (data were disposed five years post-trial according to the applicable guideline) and available literature was inconclusive and conflicting. |  |
| Murphy 2019 [73] | **Acupressure**  42 × daily sessions (6 weeks)  Daily self-administered acupressure where pressure was applied to prescribed acupoints (five acupoints in relaxing acupressure group and six in stimulating acupressure groups) in a circular motion for three minutes per point. | Usual care | n*=*4 (bruising, skin break, muscle spasm, and headache) |  | NR |
| Yeh 2016 [76] | **Auricular point acupressure (APA)**  20 × daily sessions (5 sessions/week; 4 weeks)  Vaccaria seeds were placed on prescribed ear points corresponding to the lower back. Participants pressed the seeds on each ear (1) at least three times a day for 3 min each time using the thumb and forefinger and (2) whenever they experienced pain. Participants were instructed to remove the tape and seeds after five days and be free of tape two days/week. | Sham APA (vaccaria seeds placed on ear points corresponding to stomach, mouth, duodenum, and eye) | NR |  | *Funding*: The Aging Institute of the University of Pittsburgh (UMP) Medical Center and the UMP  *CoI*: NR |
| **Multimodal Intervention** | | | | | |
| Garcia 2021 [64] | **Immersive pain relief skills virtual reality (VR) program**  56 × daily sessions (8 weeks)  An interactive 3D VR program delivered a multifaceted combination of pain relief skills training (biopsychosocial education, diaphragmatic breathing training, relaxation response exercises, and executive functioning games) through a prescribed sequence of daily immersive experiences. | Sham VR  (2D, non-immersive VR, displaying nature footage with neutral music) | n*=*12 (nausea and motion sickness) Five of these were from the control group. |  | *Funding*: AppliedVR, Inc supported the study.  *CoI*: All nine authors were associated with the AppliedVR, Inc who supported the study. Their association included being employees (n=3), the president (n=1), the chief science advisor (n=1), consultants (n=3), and a prior advisor and minor shareholder of AppliedVR, Inc (n=1). |
| **Psychological Interventions (including relaxation therapy)** | | | | | |
| Esmer 2010 [62] | **Mindfulness-based stress reduction (MBSR) therapy**  8 × 1.5-hr to 2.5-hr weekly classroom learning (8 weeks) as well as a 6-hr MBSR course at 6th week.  During the trial, participants were encouraged to meditate for 45 minutes per day with the aid of guided meditation audiotapes and complete a series of homework assignments to incorporate mindfulness into daily living. | Usual care | NR |  |  |
| Mateu 2018 [72] | **Progressive muscular relaxation (PMR)**  Crossover RCT with 2 × 4-week treatment blocks (plus 1-month washout period after each treatment block)  28 × daily sessions  Participants listened to PMR script recordings at least once per day. PRM involved a progressive tightening and relaxing of different muscle groups throughout the body. | Attention control (Relaxation music) | n*=*0 |  |  |

Note: Studies were grouped alphabetically by intervention types then by first author. Study results were omitted from meta-analyses when 1) post-intervention mean and SD were not available, 2) published data was insufficient to allow imputation, 3) contacted authors were unable to provide the necessary data, and/or 4) primary outcomes (sleep) were not included in the sleep meta-analysis. CoI=conflict of interest; Con=control group; LBP=low back pain; MOS=Medical Outcomes Study; NR=not reported; NRS=numeric rating scale; ODI=Oswestry Disability Index; PEMF=pulsed electromagnetic field; PEMS=pulsed electromagnetic signals; PENS=percutaneous electrical nerve stimulation; PROMIS=Patient-Reported Outcomes Measurement Information System; PSQI=Pittsburgh Sleep Quality Index; RCT=randomised controlled trial; RMDQ=Roland‐Morris Disability Questionnaire; RR=relative risk; r-TMS=repetitive transcranial magnetic stimulation; TENS=transcutaneous electrical nerve stimulation; VAS=visual analogue scale; VR=virtual reality.

^a^ Study author reported pooled data (manual acupuncture and electroacupuncture) as the experimental group.

^b^ Adverse events included any new medical condition or exacerbation of an existing health condition regardless of its association to the interventions and, therefore, not mentioned in detail in the current review.

^c^ Data involved imputations (e.g., imputing missing SD or post-intervention effect within group, pooling data, reversing scale).

^d^ Some data was extracted from images.

^e^ Scores cannot be reversed and combined with the sleep disturbance subscale as the information on the maximum possible scores for each subscale was not available and relevant literature was inconclusive or conflicting.^1-4^

**References for** **Appendix 4.** Details of interventions, outcome measures not included in meta-analyses, sources of funding, and conflicts of interest

1. Call-Schmidt TA, Richardson SJ. Prevalence of sleep disturbance and its relationship to pain in adults with chronic pain. Pain Manag Nurs. 2003;4(3):124-33. <https://doi.org/10.1016/S1524-9042(02)54212-0>

2. Frighetto L, Marra C, Bandali S, et al. An assessment of quality of sleep and the use of drugs with sedating properties in hospitalized adult patients. Health Qual Life Outcomes. 2004;2:17-. <https://doi.org/10.1186/1477-7525-2-17>

3. Higgins PA. Patient perception of fatigue while undergoing long-term mechanical ventilation: incidence and associated factors. Heart Lung. 1998;27(3):177-83. <https://doi.org/10.1016/S0147-9563(98)90005-X>

4. Snyder-Halpern R, Verran J. Instrumentation to describe subjective sleep characteristics in healthy subjects. Res Nurs Health. 1987;10(3):155-63. <https://doi.org/10.1002/nur.4770100307>

Appendix 5: Data handing

Authors have been contacted for missing data. Where responses were not received or insufficient data remained available, we handled the data as per the process below. Cochrane formulae were used when imputing SD from SE, p-values, or confidence intervals.^a^

^a^ Higgins JP, Li T, Deeks JJ. Chapter 6: Choosing effect measures and computing estimates of effect. 2021. In: Cochrane Handbook for Systematic Reviews of Interventions [Internet]. Cochrane. Available from: <https://training.cochrane.org/handbook/current/chapter-06>.

| **Study** | **Outcome** | **Data handling** |
| --- | --- | --- |
| Carlsson 2001 [60] | [P] VAS-100 | - Post-intervention mean and SD for morning and evening measures were extracted from image. - Morning and evening post intervention mean and SD were pooled for each group. |
| de Teresa 2021 [61] | [S] MOS - Sleep | A composite score was not reported. Scores for individual questionnaire items were pooled to provide a composite score for the meta-analysis. |
|  | [P] VAS-10 | - Baseline mean and post-intervention SD were extracted from image. - Post-intervention SD was calculated from 95% CI for each group. |
| Esmer 2010 [62] | [S] Abridged PSQI  [P] Summary VAS  [D] RMDQ | - Post-intervention mean was calculated from baseline and change from baseline data. - Baseline SD was used as post-intervention SD. |
|  | [S] Abridged PSQI | Post-intervention mean was reversed for meta-analysis to ensure high scores reflected poorer health outcomes. |
| Field 2007 [63] | Overall | N analysed for each group was not reported. N randomised was used. |
| Garcia 2021 [64] | [S] PROMIS – Sleep disturbance (6a)  [S] PROMIS – Physical function (6b) | Unpublished raw mean and SD as provided by the trial author were used in meta-analysis. |
|  | [P] DVPRS | - 95% CI was extracted from image - Post-intervention SD was calculated from 95% CI for each group. |
| Ghoname 1999a [65] | Overall | N analysed for each group was not reported. N randomised was used. |
| Ghoname 1999b [66] | Overall | N analysed for each group was not reported. N randomised was used. |
| Ghoname 1999c [67] | Overall | N analysed for each group was not reported. N randomised was used. |
|  | [S] VAS-10 (sleep quality)  [P] VAS-10 | Data from all experimental arms (4, 15/30, 100hz) were pooled. |
|  | [S] VAS-10 | Post-intervention mean and SD for each group were extracted from image. |
| Hamza 1999 [69] | Overall | N analysed for each group was not reported. N randomised was used. |
|  | [S] VAS-10 | Mean percentage of change in each group post-intervention was extracted from image. |
|  | [P] VAS-10 | Data from all experimental arms (15/30/45 min) were pooled. |
| Hagiwara 2017 [68] | [S] AIS  [P] VAS-10  [D] RMDQ | - Post-intervention mean was calculated from baseline and change from baseline data. - Baseline SD was used as post-intervention SD. |
| Hernandez-reif 2001 [70] | Overall | N analysed for each group was not reported. N randomised was used. |
| Murphy 2019 [73] | [S] PSQI  [D] RMDQ | - Post-intervention mean and 95%CI were extracted from image. - Post-intervention SD was calculated from 95% CI. - Data from two active intervention arms (relaxing acupressure and stimulating acupressure) were pooled prior to inclusion in meta-analysis. |
| Licciardone 2020 [71] | [S] PROMIS 29  [P] NRS-10  [D] RMDQ | - Post-intervention mean was calculated from baseline and change from baseline data. - Baseline SD was used as post-intervention SD |
| Mateu 2018 [72] | Overall | N analysed for each group was calculate form the percentage of patients in each group |
|  | [S] MOS Sleep (SLP9)  [P] VAS-10 | Post intervention mean and SD for mild, moderate, and severe groups were pooled. |

Note: [S]=sleep; [P]=pain intensity; [D]=back-related disability. AIS=Athens Insomnia Scale; BPI-sf=Brief Pain Inventory–short form; DVPRS=Defense and Veterans Pain Rating Scale; MOS=Medical Outcomes Study; NRS=numeric rating scale; PROMIS=Patient-Reported Outcomes Measurement Information System; PSQI=Pittsburgh Sleep Quality Index; RMDQ=Roland‐Morris Disability Questionnaire; VAS=visual analogue scale.

Appendix 6: Summary of amendments to information provided at registration or in the protocol.

| Information at registration or in protocol | Amendments |
| --- | --- |
| Post-intervention mean and SD would be used as the measure of effect | Where post-intervention data could not be imputed for a study, mean change from baseline and SD were used in the meta-analysis. Combining follow-up and change data is valid in meta-analysis as there are no relevant differences between follow-up and change data standardised mean differences (standardised mean differences are scattered around the null)^a^ |
| Publication bias would be assessed via visual inspection of funnel plots and the Egger’s test | Clarification was added to specify that publication bias was assessed via visual inspection of funnel plots and, where there were ≥10 studies, the Egger’s test. |

^a^ da Costa BR, Nüesch E, Rutjes AW, Johnston BC, Reichenbach S, Trelle S, et al. Combining follow-up and change data is valid in meta-analyses of continuous outcomes: A meta-epidemiological study. J Clin Epidemiol 2013;66(8):847-55. <https://doi.org/10.1016/j.jclinepi.2013.03.009>

Appendix 7: Measurement instruments used in included studies

| **Measurement instrument** | **Short name** | **Type of measurement** | **Brief description** | **Score range** |
| --- | --- | --- | --- | --- |
| **Sleep Measurement** | | | | |
| Abridged Pittsburgh Sleep Quality Index [1] | Abridged PSQI | Self-reported | A 5-item yes/no questionnaire assessing five aspects of sleep: sleep quality, insomnia medication use, time until onset of sleep, duration of nightly sleep, and restoration after sleep. Higher scores indicate better sleep quality.  *Note*: For the current review, the score for Abridge PSQI was converted before pooling to ensure low scores indicate better sleep quality. | 0-5 |
| Athens Insomnia Scale [2] | AIS | Self-reported | An 8-item questionnaire assessing the severity of insomnia. Each item is rated 0‑3 (0 = no problem at all; 3 = very serious problem). | 0-24 |
| Medical Outcomes Study Sleep [3, 4] | MOS | Self-reported | A 12-item questionnaire assessing six domains of sleep: sleep disturbance, sleep adequacy, sleep quantity, somnolence, snoring, and awakening due to short of breath or with headache. The Sleep Problem summary index (SLP9) is calculated based on nine items and re-scaled to 0-100. Higher scores for SLP9 indicates worse sleep problem.  The score for the sleep quantity item indicates the actual number of hours sleeping each night.  *Note*: In de Teresa (2021) [5], the author has confirmed via correspondence that higher scores indicate better sleep outcomes except for the parameters “Time to fall asleep", "Enough sleep to feel rested" and "Amount of sleep needed" with which the opposite would occur (i.e., lower scores indicate better sleep outcomes). This direction of score differed from the standard MOS scores in which higher scores indicate worse sleep outcomes. For the current review, MOS scores from this RCT was converted before pooling to ensure higher scores indicate worse sleep outcomes. | SLP9  0-100 |
| Patient-Reported Outcomes Measurement Information System – Sleep Disturbance Short Form (version 6a; Northwestern University, n.d.) [6] | PROMIS (6a) | Self-reported | A 6-item short form assessing sleep disturbance (difficulties getting to sleep or staying asleep, sleep adequacy, and satisfaction with sleep) over the past seven days (1 = very good; 5 = very poor). | 6-30 |
| Patient-Reported Outcomes Measurement Information System with 29 items (version unconfirmed; Northwestern University, n.d.) | PROMIS-29 | Self-reported | A 29-item adult profile instrument evaluating physical, mental, and social health in adults, including a 4-item Sleep Disturbance component. Each of sleep disturbance item is scored 1-5 points (1 = very good; 5 = very poor) and raw scores (4-20) are converted to a T‑score (32-73.3) with a standardised mean of 50 and a standard deviation of 10 (as calibrated against American general population). Higher scores indicate poorer health outcomes. | 32-73.3 |
| Pittsburgh Sleep Quality Index [7] | PSQI | Self-reported | A 19-item questionnaire assessing seven components of sleep: sleep quality, sleep latency, duration, efficiency, disturbances, use of sleeping medications, and daily dysfunction within the past month. Each component is scored 0-3. Higher scores indicate worse sleep quality. | 0-21 |
| Sleep diary [8] | - | Self-reported | Sleep quality was scored according to a three-category scale (good,  slightly disturbed by pain and woke 1 or 2 times, or badly disturbed by pain and woke more than twice). | NR |
| Sleep Scale [9] | - | Self-reported | A 15-item scale rated on a visual analogue anchored at one end with effective sleep responses and at the opposite end with ineffective responses. Each item is scored 0-10. The scale contains three subscales: sleep disturbance, sleep effectiveness, and supplementary sleep (e.g., naps). Higher scores indicate worse symptoms for the sleep disturbance subscale, but better sleep for sleep effectiveness, and supplementary sleep subscales. | 0-150 |
| Visual analogue scale (for sleep quality) | VAS-10 | Self-reported | A unidimensional measure of an outcome (e.g., sleep quality). It comprises of a straight line usually 10cm in length anchored by two descriptors one for each symptom extreme e.g., for sleep quality: 0 = best sleep quality; 10 = worst sleep quality. [10] | 0-10 |
| **Pain Intensity Measurement** | | | | |
| Brief Pain Inventory-short form [11] | BPI-sf | Self-reported | A questionnaire assessing pain intensity and pain interferences during the previous seven days. The pain intensity subscale comprises four items (i.e., pain at its worst, least, average, and now; 0 = no pain; 10 = pain as bad as you can imagine). |  |
| Defense and Veterans Pain Rating Scale [12] | DVPRS | Self-reported | An 11-point numeric rating scale measuring average pain intensity over the previous 24 hours (0 = no pain; 10 = as bad as it could be and nothing else matters). | 0-10 |
| Numeric rating scale | NRS-10 | Self-reported | A unidimensional measure of a symptom (e.g., pain intensity). Respondents are to select a whole number (0-10) that best reflects their pain intensity (e.g., 0 = no pain; 10 = worst pain. [13] | 0-10 |
| Summary VAS [1] | - | Self-reported | The scale is consisted of three Likert-scale surveys measuring average pain, worst pain, and comparison of pain present this week vs. last week (0 = no pain; 10 = worst pain imaginable). | 0-30 |
| Visual analogue scale (for pain intensity) | VAS-10  VAS-100 | Self-reported | A unidimensional measure of a symptom (e.g., pain intensity or sleep disturbance). It comprises of a straight line usually 10cm in length anchored by two descriptors one for each symptom extreme e.g., for pain intensity: 0 = no pain; 10 (or 100) = worst imaginable pain.^13^ VAS-10 indicates the score range of 0-10. VAS-100 indicates the score range of 0-100. | 0-10  or  0-100 |
| **Back Disability Measurement** | | | | |
|  | | | | |
| Patient-Reported Outcomes Measurement Information System – Physical Function Short Form (version 6a; Northwestern University, n.d.) | PROMIS (6b) | Self-reported | A 6-item short form assessing physical function (e.g., difficulties walking, going up/down stairs, doing moderate housework) (1 = unable to do; 5 = without any difficulty). | 6-30 |
| Oswestry Disability Index [14, 15] | ODI | Self-reported | A 10-item questionnaire assessing pain-related disability in persons with LBP. Each item (pain intensity, personal care, lifting, walking, sitting, standing, sleeping, social life, traveling, and employment/homemaking) is scored 0-5 points. The total (up to 50) is doubled to create a global score of up to 100.  Statements in the pain intensity section ranges from ‘no pain’ to ‘worst imaginable pain’. In the sleeping section, statements range from ‘sleep never disturbed by pain’ to ‘pain prevents me from sleeping at all’. | 0-100 |
| Roland‐Morris Disability Questionnaire [16] | RMDQ | Self-reported | A 24-item yes/no questionnaire assessing LBP function and disability with higher scores indicating a more severe degree of disability. | 0-24 |

**References for Appendix 7.** Measurement instruments used in included studies

1. Esmer G, Blum J, Rulf J, et al. Mindfulness-based stress reduction for failed back surgery syndrome: a randomized controlled trial. J Osteopath Med. 2010;110(11):646-52. <https://doi.org/10.7556/jaoa.2010.110.11.646>

2. Soldatos C, Dikeos D, Paparrigopoulos T. Athens Insomnia Scale: validation of an instrument based on ICD-10 criteria. J Psychosom Res. 2000;48(6):555-60. <https://doi.org/10.1016/S0022-3999(00)00095-7>

3. Hays R, Martin S, Sesti A, et al. Psychometric properties of the Medical Outcomes Study Sleep measure. Sleep Med. 2005;6(1):41-4. <https://doi.org/10.1016/j.sleep.2004.07.006>

4. Hays R, Stewart A. Sleep Measures. In: Stewart A, Ware J, editors. Measuring functioning and well-being: the medical outcomes study approach: Duke University Press; 1992. p. 235–59.

5. de Teresa C, Varela-López A, Rios-Álvarez S, et al. Evaluation of the analgesic efficacy of a bioelectronic device in non-specific chronic low back pain with neuropathic component. A randomized trial. J Clin Med. 2021;10(8):1781. <https://doi.org/10.3390/jcm10081781>

6. Yu L, Buysse D, Germain A, et al. Development of short forms from the PROMIS™ Sleep Disturbance and Sleep-Related Impairment item banks. Behav Sleep Med. 2012;10(1):6-24. <https://doi.org/10.1080/15402002.2012.636266>

7. Buysse DJ, Reynolds CF, Monk TH, et al. The Pittsburgh Sleep Quality Index: a new instrument for psychiatric practice and research. Psychiatry Res. 1989;28(2):193-213. <https://doi.org/10.1016/0165-1781(89)90047-4>

8. Carlsson CP, Sjölund BH. Acupuncture for chronic low back pain: a randomized placebo-controlled study with long-term follow-up. Clin J Pain. 2001;17(4):296-305. <https://doi.org/10.1097/00002508-200112000-00003>

9. Snyder-Halpern R, Verran J. Instrumentation to describe subjective sleep characteristics in healthy subjects. Res Nurs Health. 1987;10(3):155-63. <https://doi.org/10.1002/nur.4770100307>

10. Ghoname E-SA, White PF, Ahmed HE, et al. Percutaneous electrical nerve stimulation: an alternative to TENS in the management of sciatica. Pain. 1999;83(2):193-9. <https://doi.org/10.1016/S0304-3959(99)00097-4>

11. Cleeland CS, Ryan KM. Pain assessment: global use of the Brief Pain Inventory. Ann Acad Med Singap. 1994;23(2):129-38.

12. Buckenmaier C, Galloway K, Polomano R, et al. Preliminary validation of the Defense and Veterans Pain Rating Scale (DVPRS) in a military population. Pain Medicine. 2013;14(1):110-23. <https://doi.org/10.1111/j.1526-4637.2012.01516.x>

13. Hawker G, Mian S, Kendzerska T, et al. Measures of adult pain: visual Analog Scale for Pain (VAS Pain), Numeric Rating Scale for Pain (NRS Pain), McGill Pain Questionnaire (MPQ), Short‐Form McGill Pain Questionnaire (SF‐MPQ), Chronic Pain Grade Scale (CPGS), Short Form‐36 Bodily Pain Scale (SF‐36 BPS), and Measure of Intermittent and Constant Osteoarthritis Pain (ICOAP). Arthritis Care Res. 2011;63(S11):S240-S52. <https://doi.org/10.1002/acr.20543>

14. Fairbank J, Couper J, Daies J, et al. The Oswestry low back pain questionnaire. Physiotherapy. 1980;66(8):271-3.

15. Fairbank J, Pynsent P. The Oswestry Disability Index. Spine. 2000;25(22):2940-53. <https://doi.org/10.1097/00007632-200011150-00017>

16. Roland M, Morris R. A study of the natural history of back pain. Part I: development of a reliable and sensitive measure of disability in low-back pain. Spine. 1983;8(2):141-4.

Appendix 8: Risk of Bias Assessment

sFig. 8.1. Risk of bias assessment for included studies (by the number of studies)


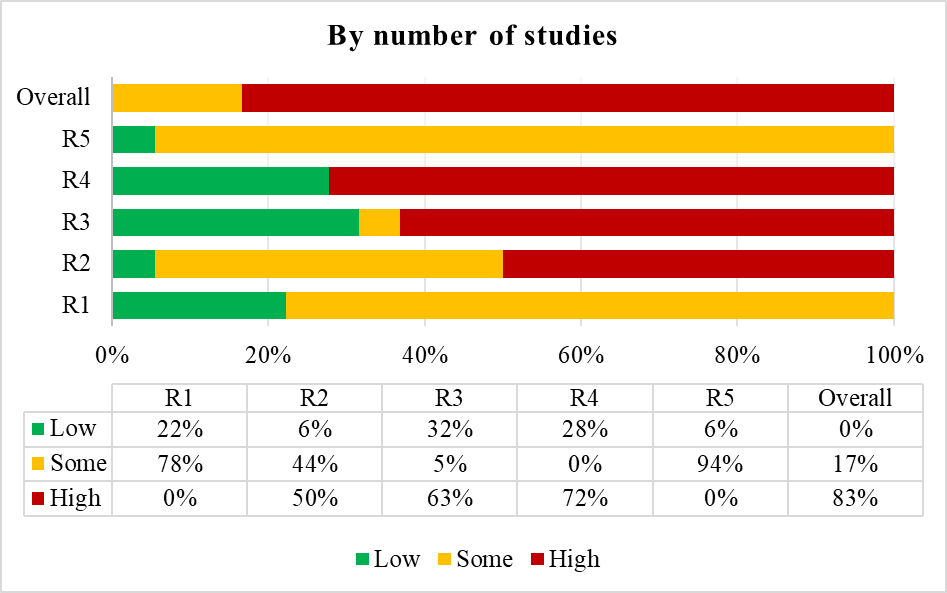


Note: R1=Randomization process; R2=Deviations from the intended interventions (assignment); R3=Missing outcome data; R4=Measurement of the outcome; R5=Selection of the reported result; Overall=Overall risk of bias for each study.

sFig. 8.2. Risk of bias assessment for included studies (by number of participants)


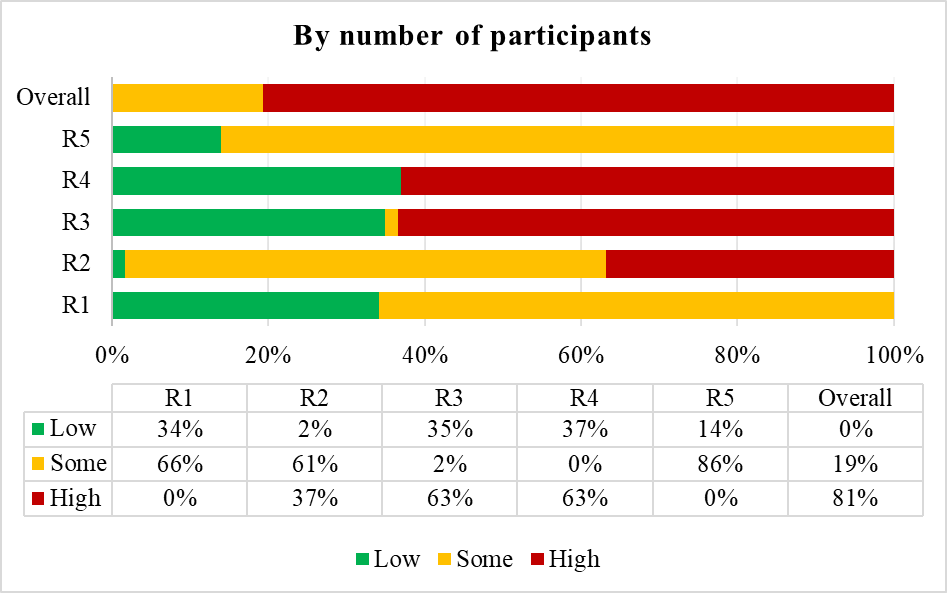


Note: Summary of the risk of bias assessment by the number of participants. R1=Randomization process; R2=Deviations from the intended interventions (assignment); R3=Missing outcome data; R4=Measurement of the outcome; R5=Selection of the reported result; Overall=Overall risk of bias for each study.

Appendix 9: ****Sensitivity analysis and funnel plot for sleep outcomes****

****sFig. 9.1. Outlier analysis for sleep****


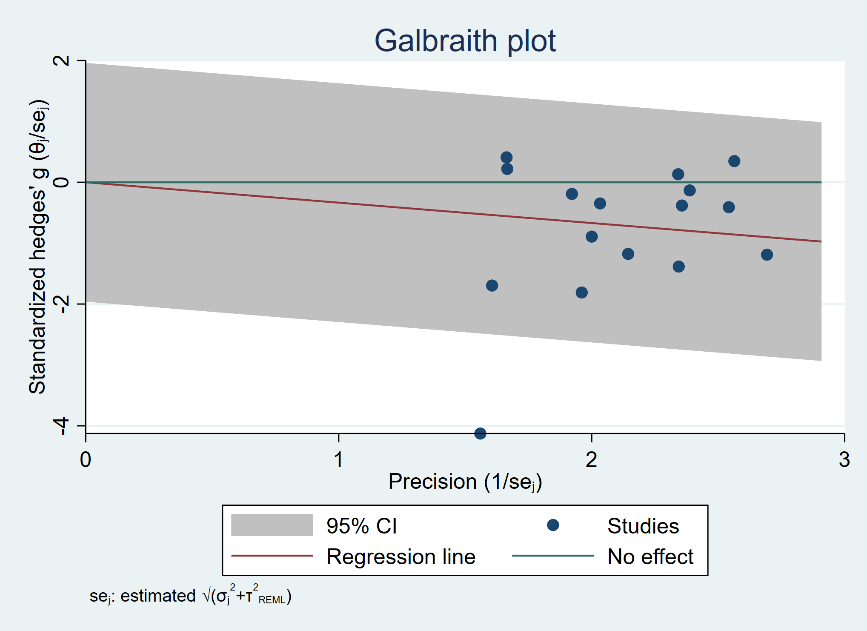


**sFig. 9.2. Influential study analysis (leave-one-out) for sleep**
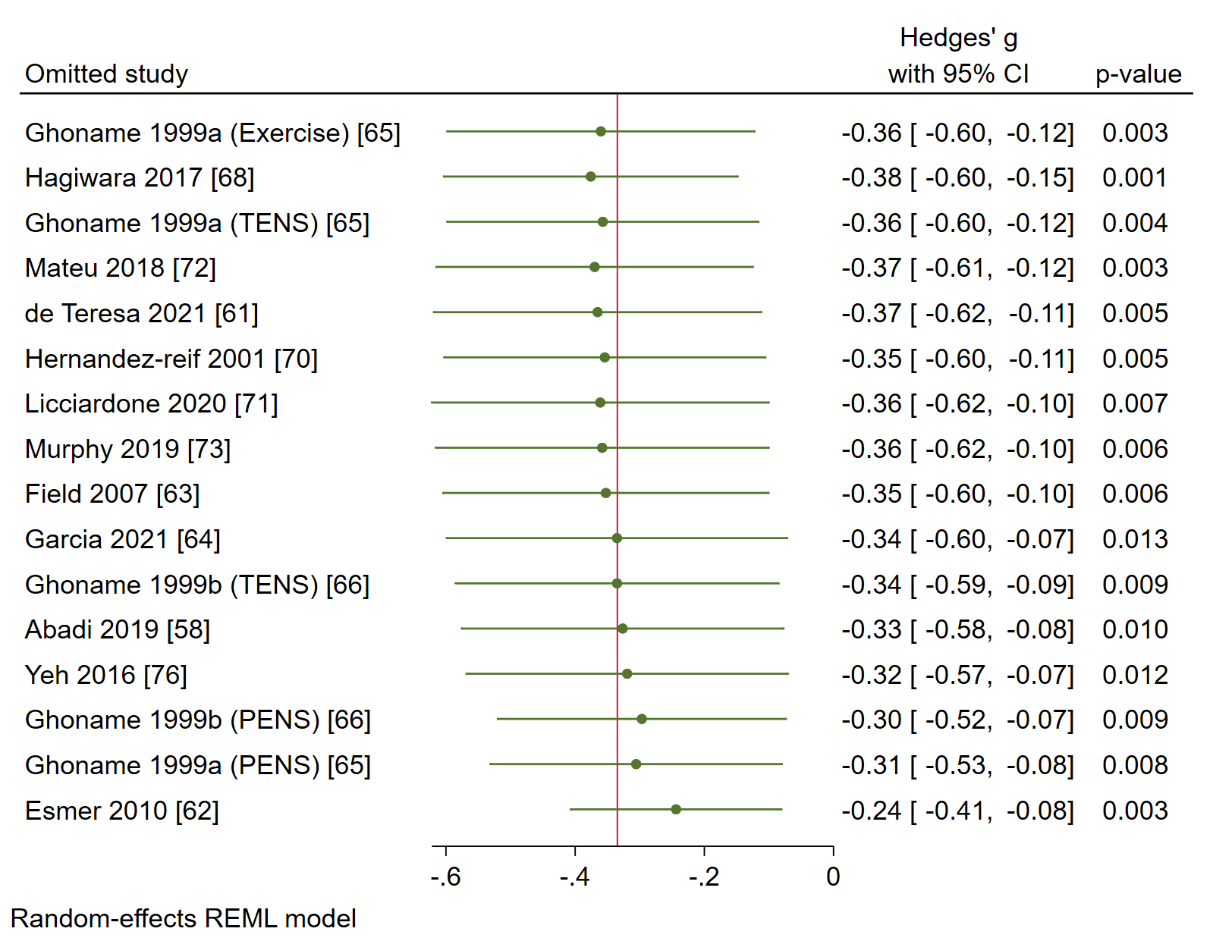


****sFig. 9.3. Funnel plot for sleep****


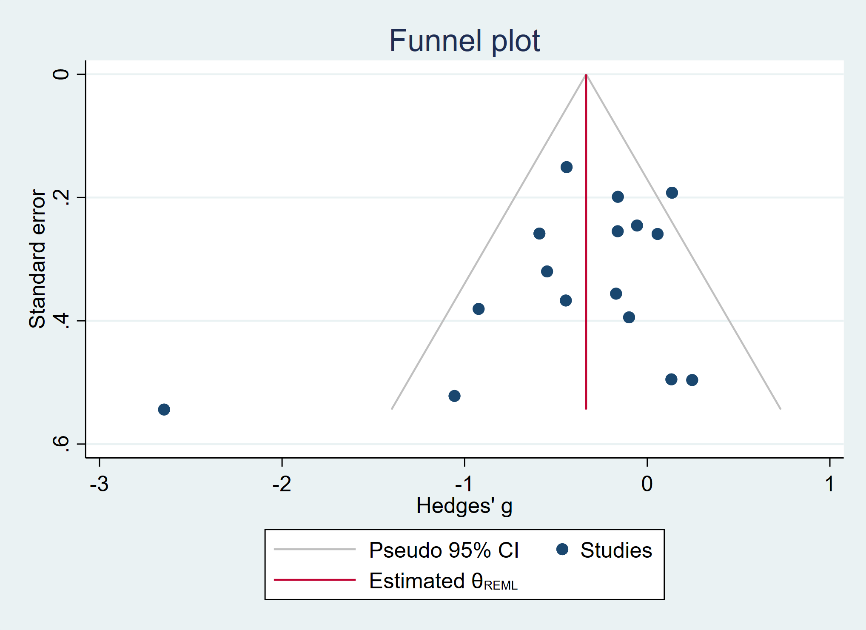


****sFig. 9.4. Sensitivity analysis –pairwise analysis for sleep with crossover RCTs excluded****


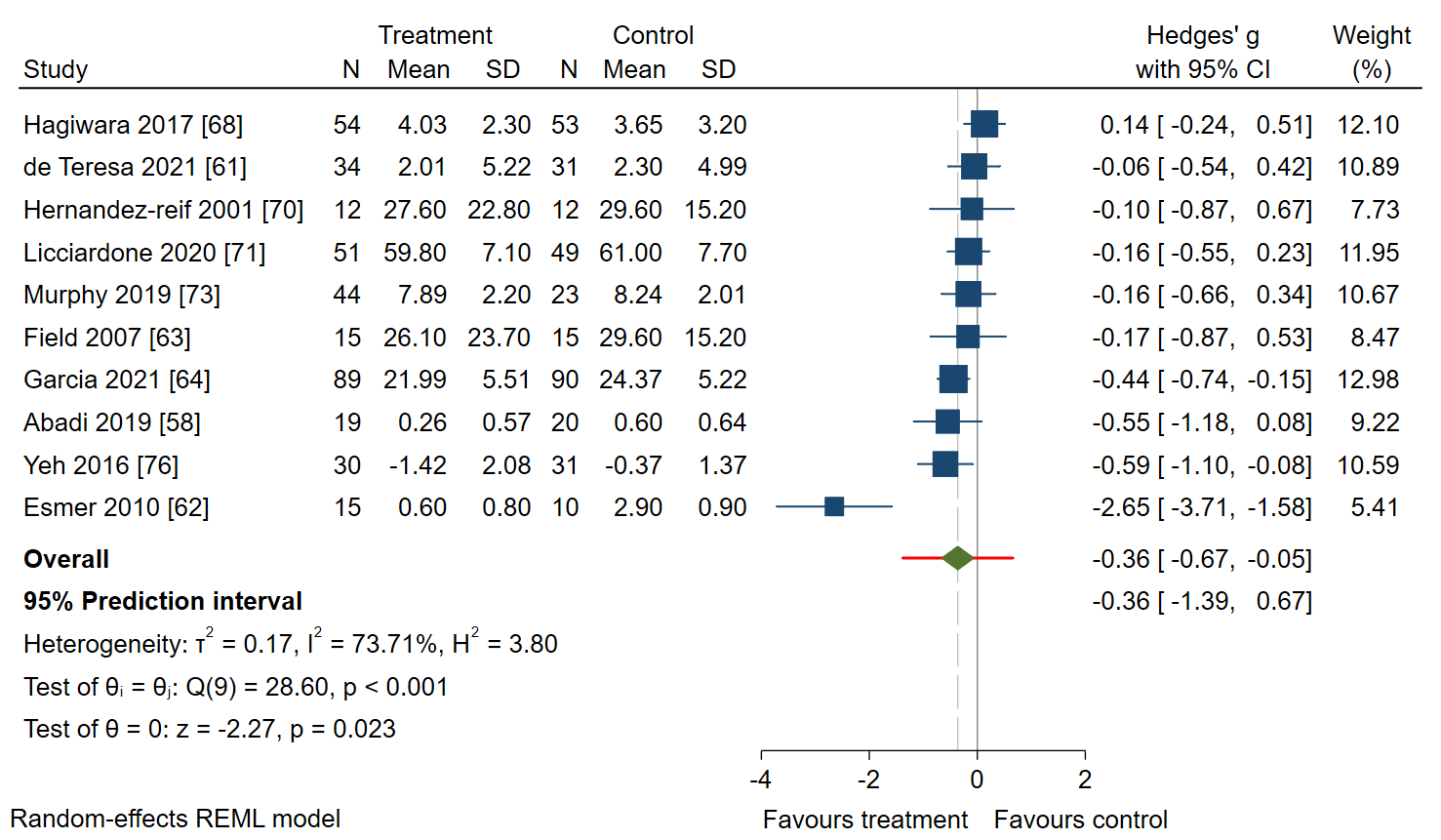


**sFig. 9.5. Influential study analysis (leave-one-out) for sleep questionnaires**


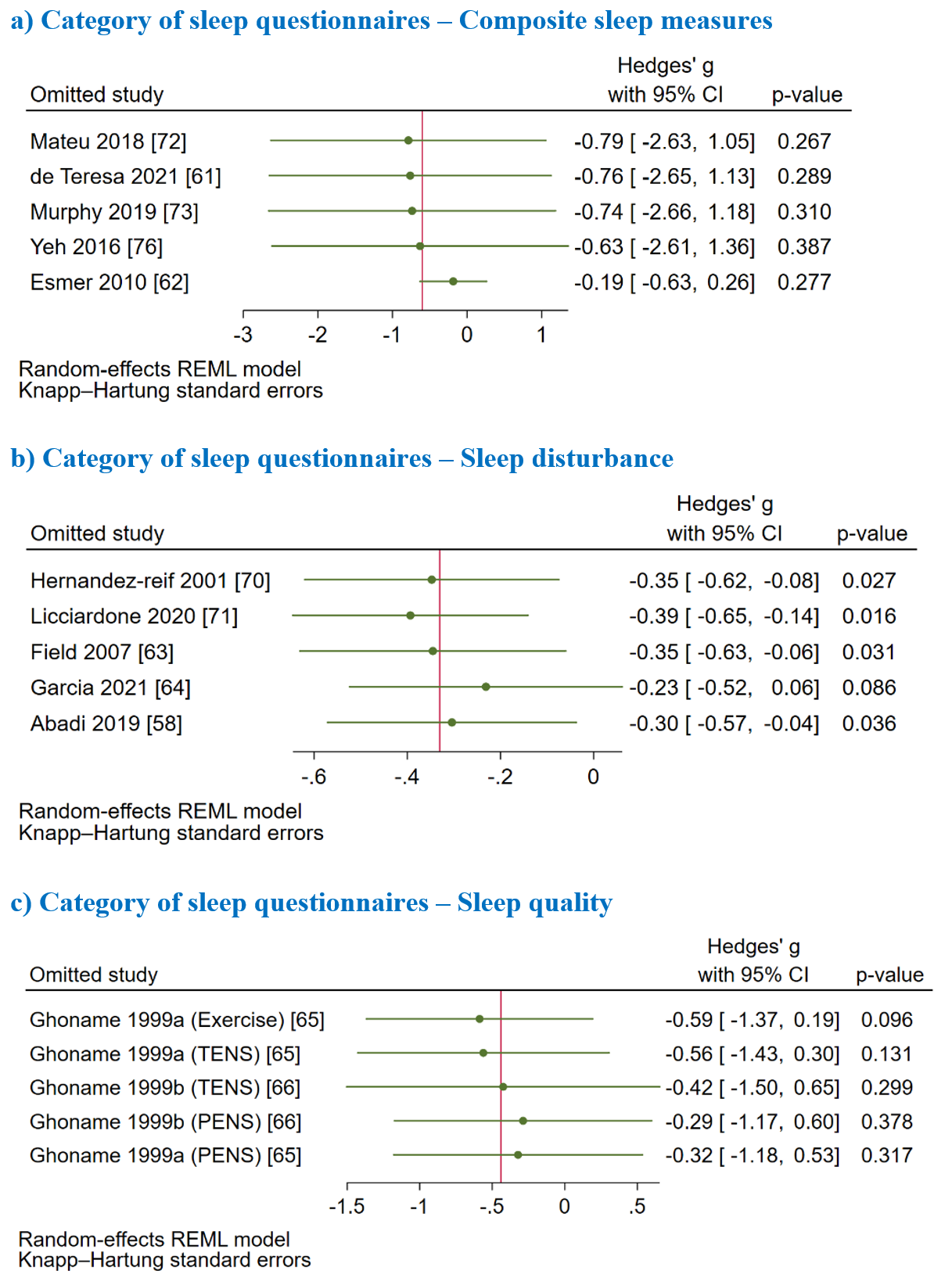


**sFig. 9.6. Outlier analysis for sleep questionnaires (Category of sleep questionnaires – Composite sleep measures)**


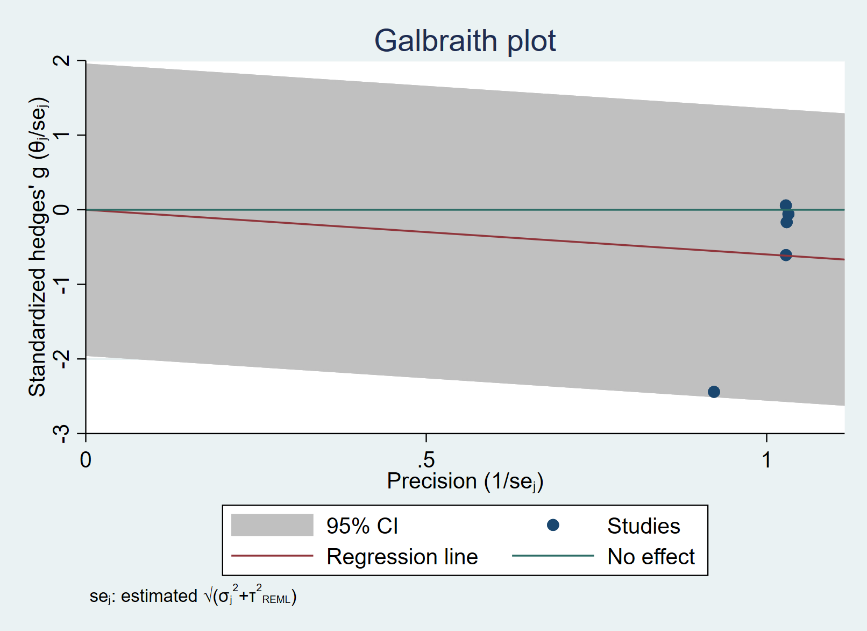


**Note: Outlier analysis was not performed for other categories of sleep questionnaires (i.e., Sleep disturbance and Sleep quality) as I^2^ <40%.**

****Appendix 10: Sensitivity analysis and funnel plot for pain intensity****

****sFig. 10.1. Outlier analysis for pain intensity****


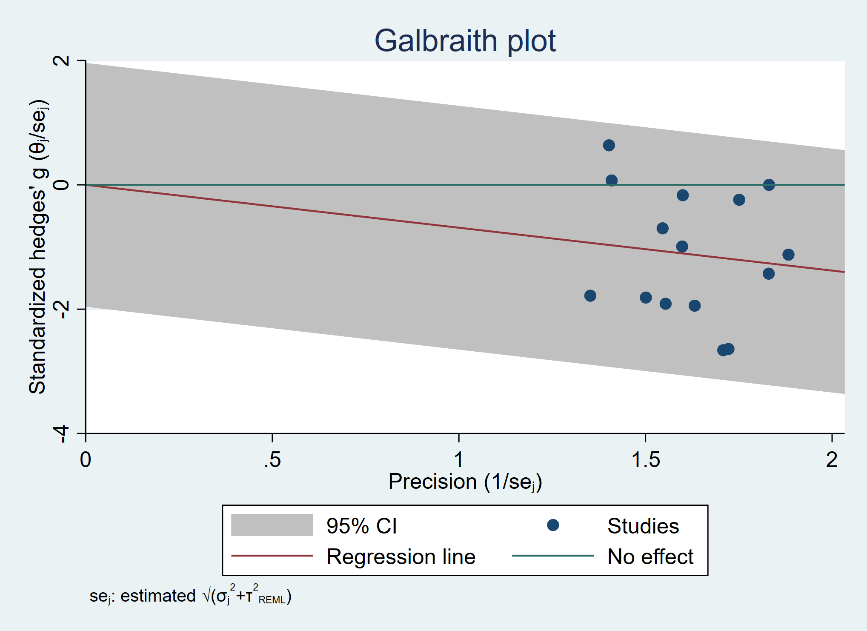


**sFig. 10.2. Influential study analysis (leave-one-out) for pain intensity**
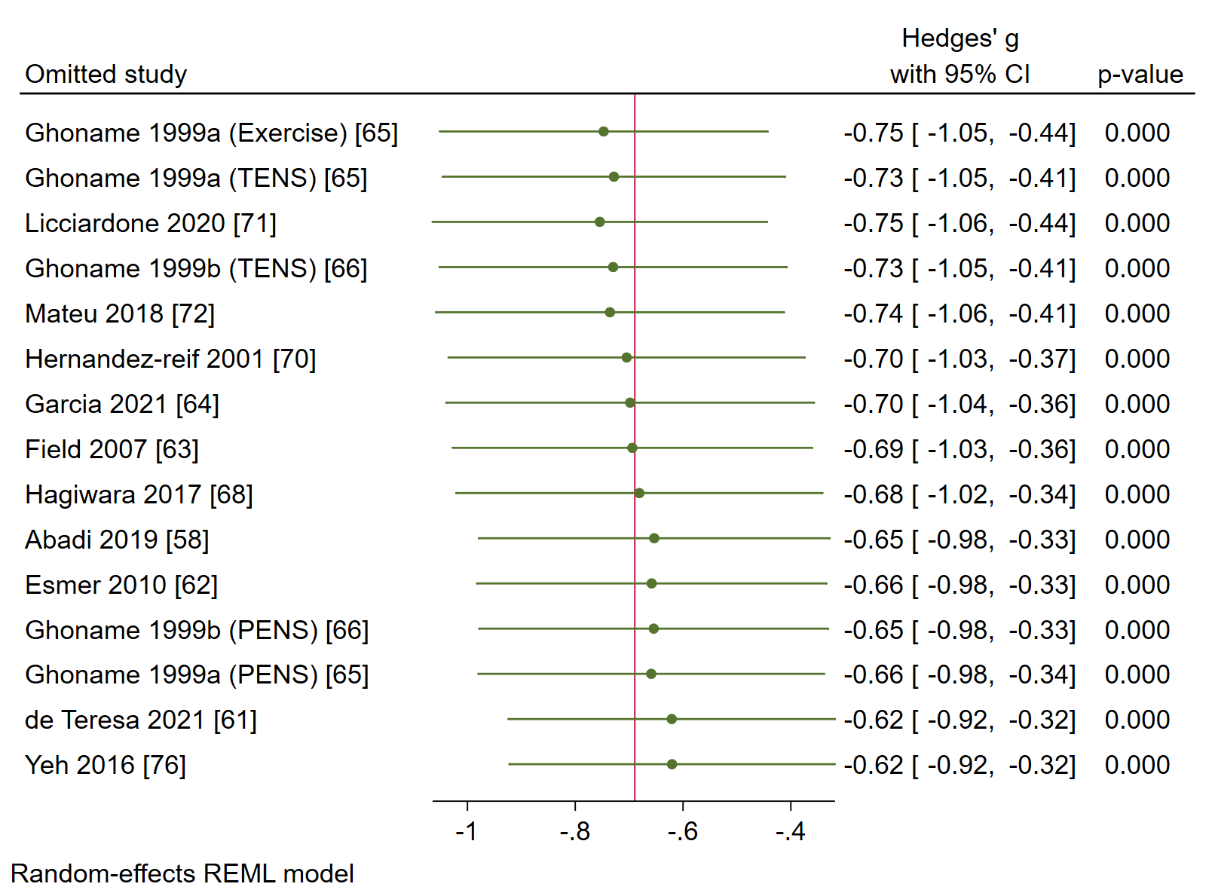


****sFig. 10.3. Sensitivity analysis –pairwise analysis for pain intensity with crossover RCTs excluded****


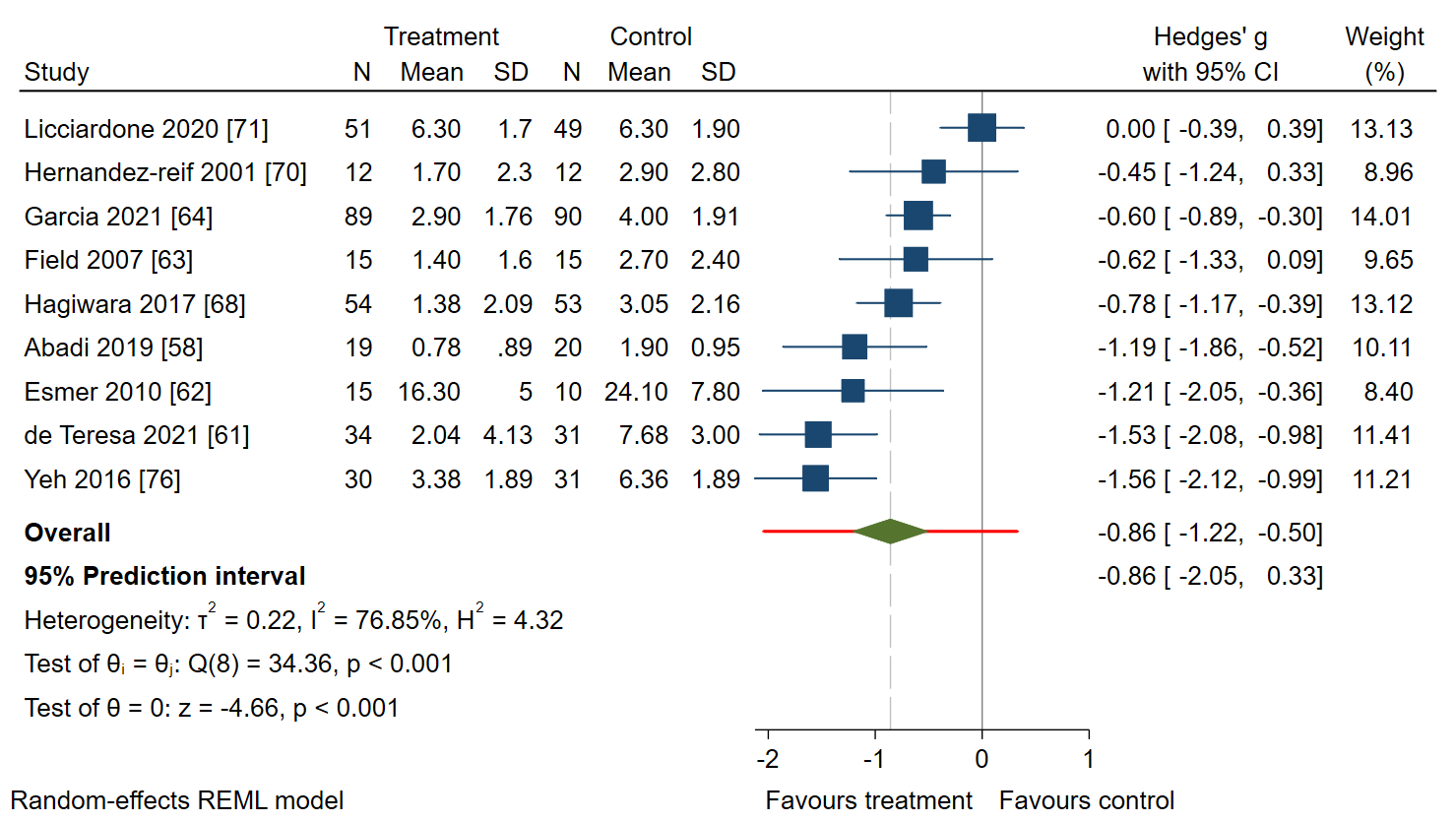


****sFig. 10.4. Funnel plot for pain intensity****


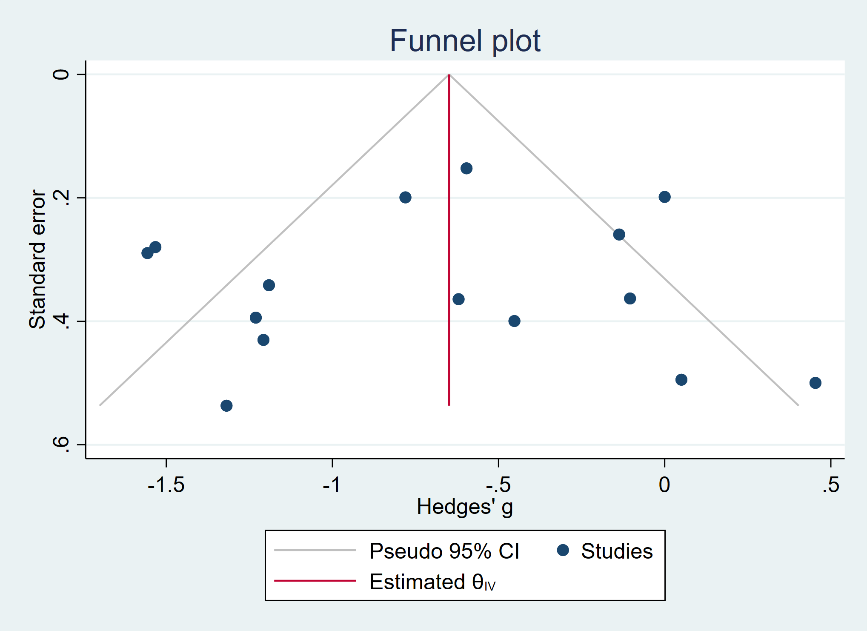


****Appendix 11: Sensitivity analysis for back-related disability****

****sFig. 11.1. Outlier analysis for back-related disability****


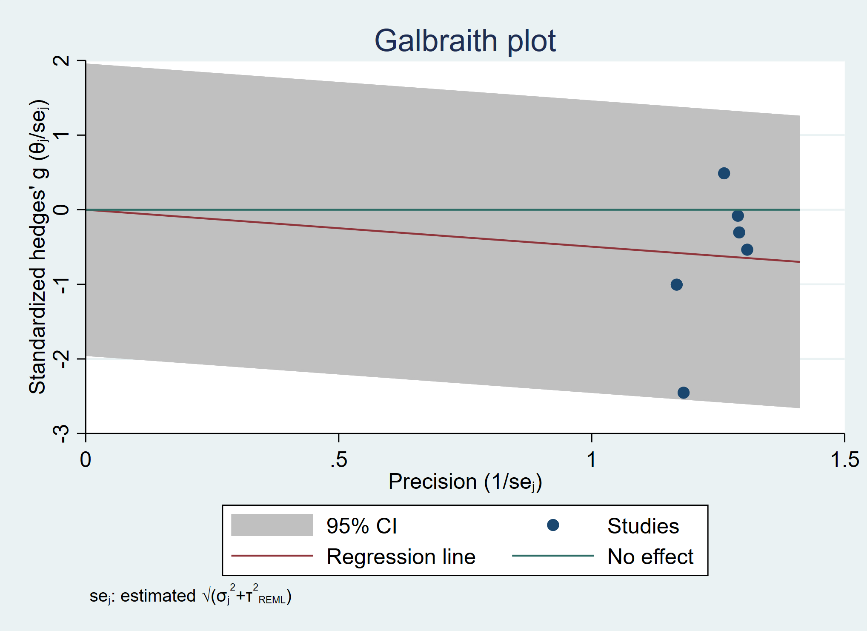


**sFig. 11.2. Influential study analysis (leave-one-out) for back-related disability**
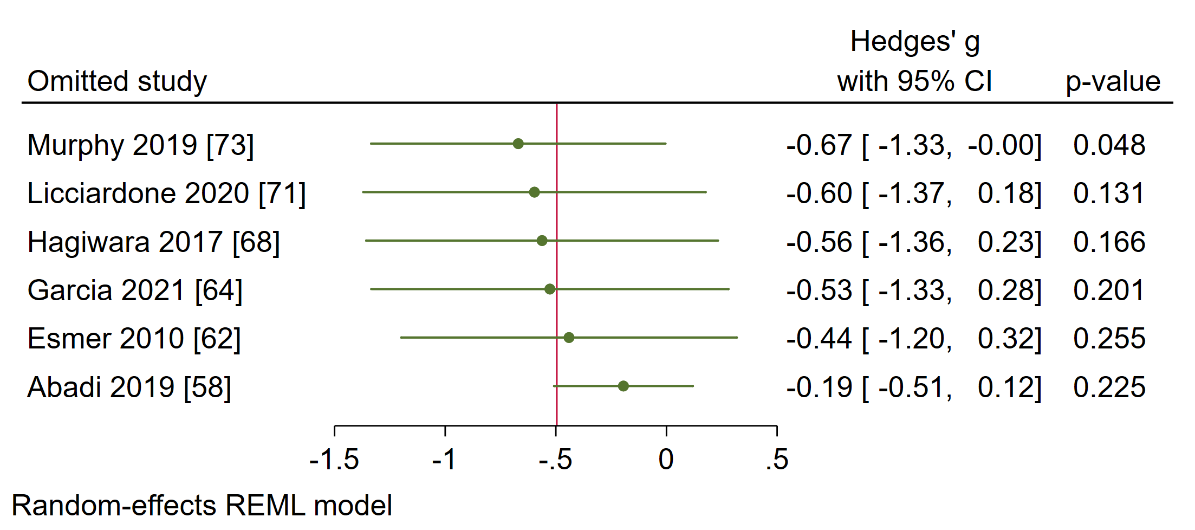


****sFig. 11.3. Funnel plot for back-related disability****


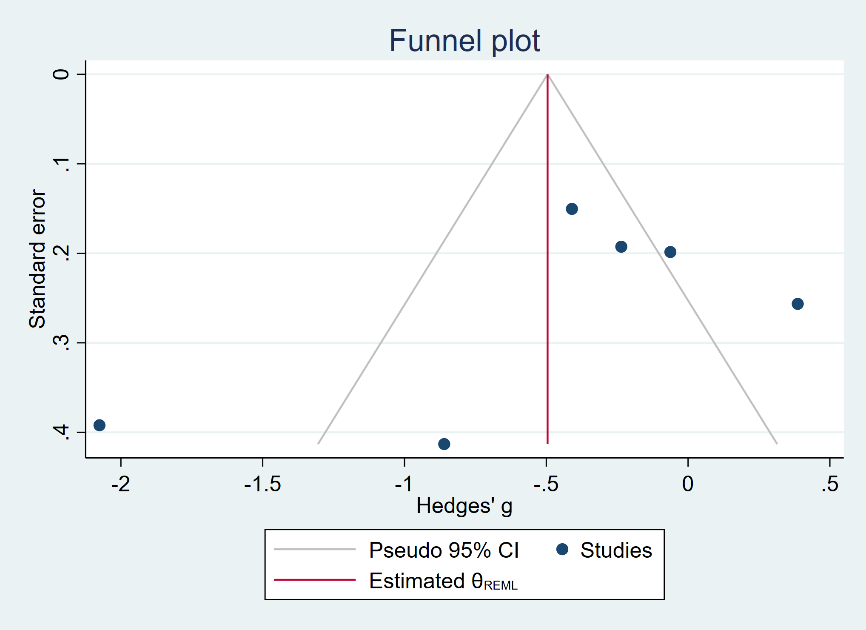


****Appendix 12: Subgroup analysis (non-specific chronic low back pain)****

****sFig. 12.1. Effect of non-pharmacological interventions on sleep in non-specific chronic low back pain****


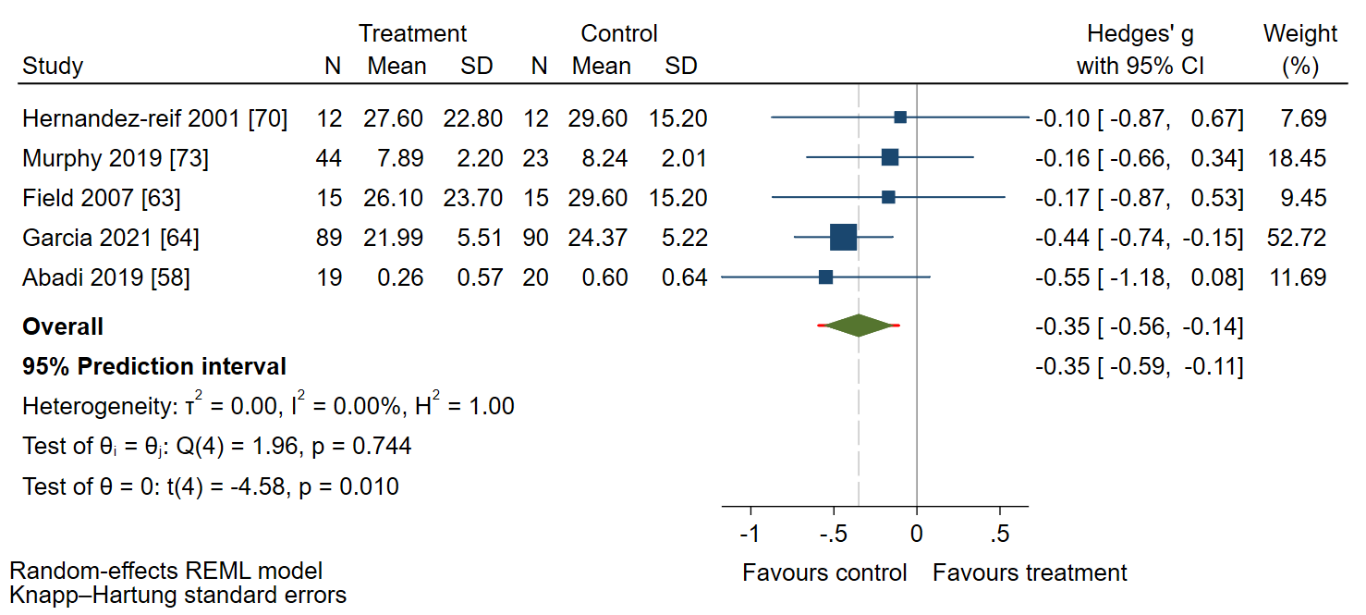


**sFig. 12.2. Effect of non-pharmacological interventions on pain intensity in non-specific chronic low back pain**
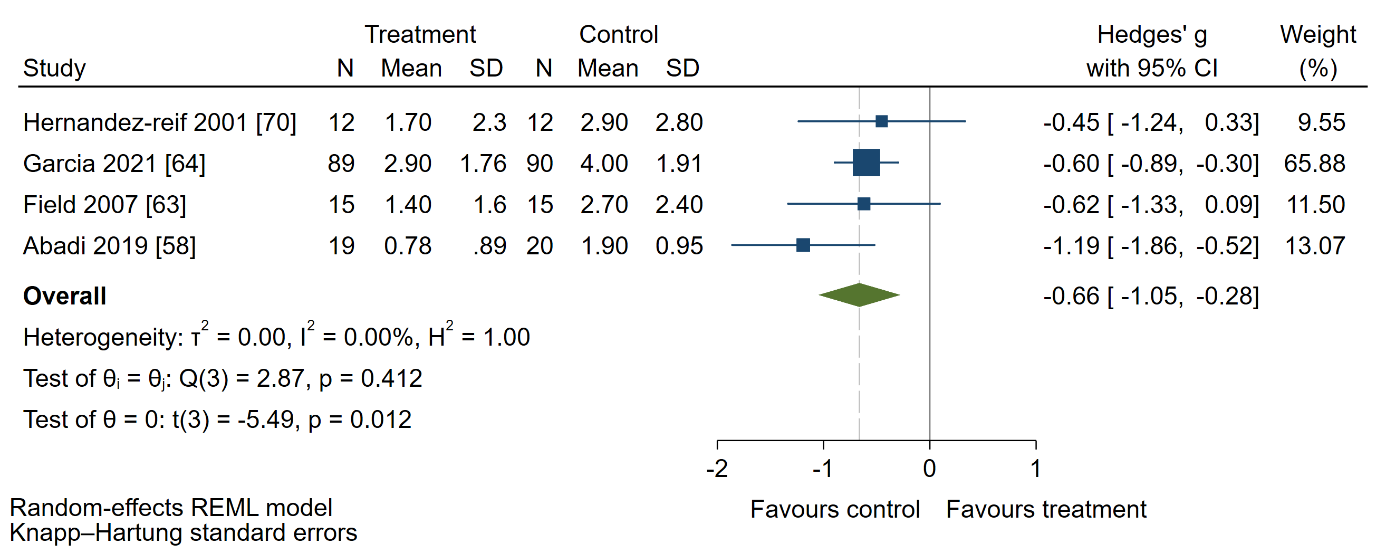


****sFig. 12.3. Effect of non-pharmacological interventions on back-related disability in non-specific chronic low back pain****


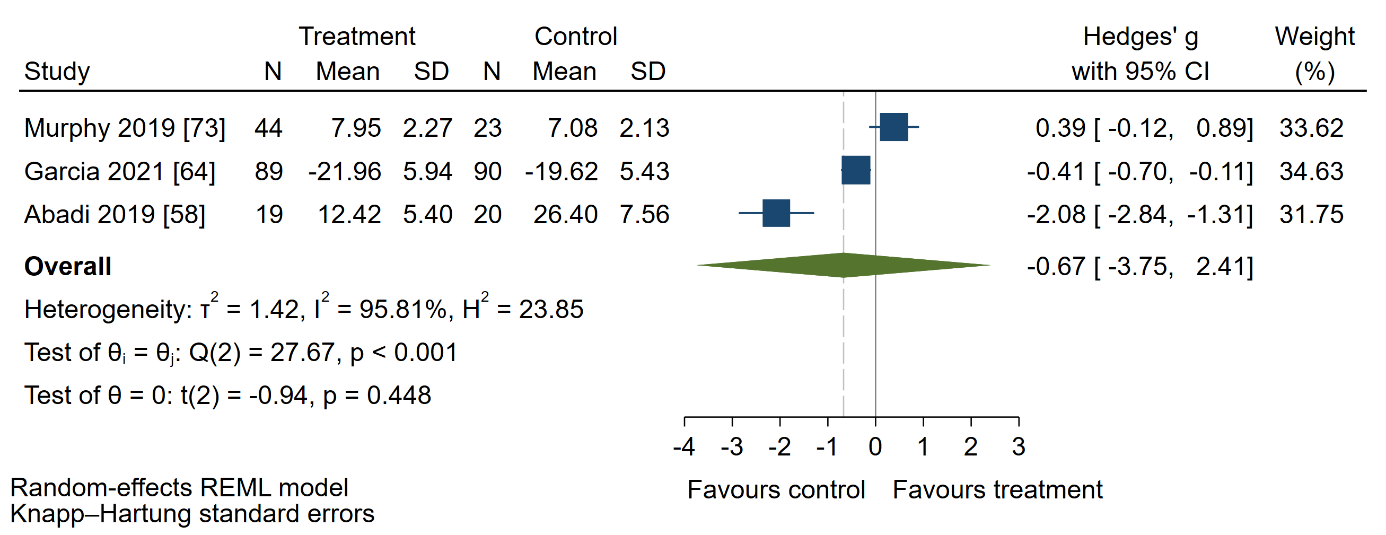


****Appendix 13: Subgroup analysis (sham treatment)****

****sFig. 13.1. Effect of non-pharmacological interventions vs. sham treatment on sleep****


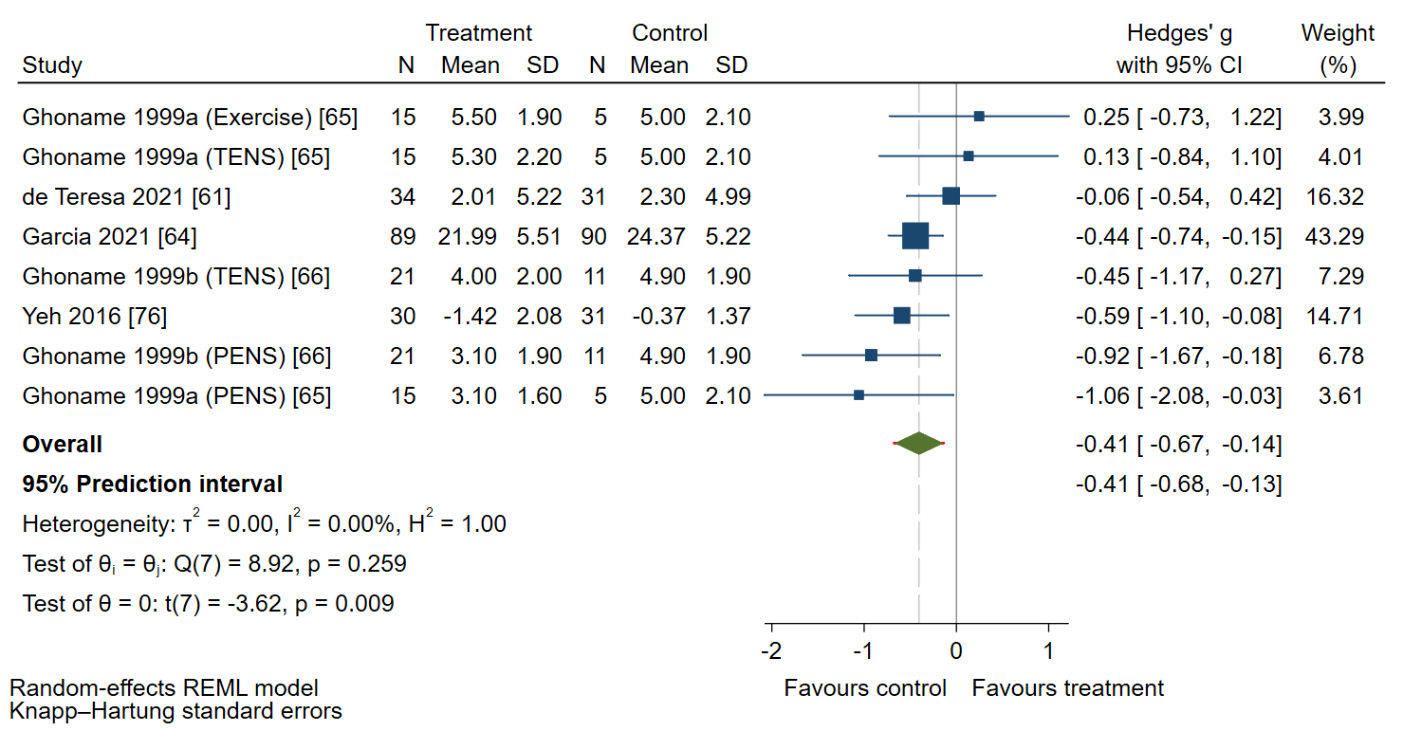


**sFig. 13.2. Effect of non-pharmacological interventions vs. sham treatment on pain intensity**
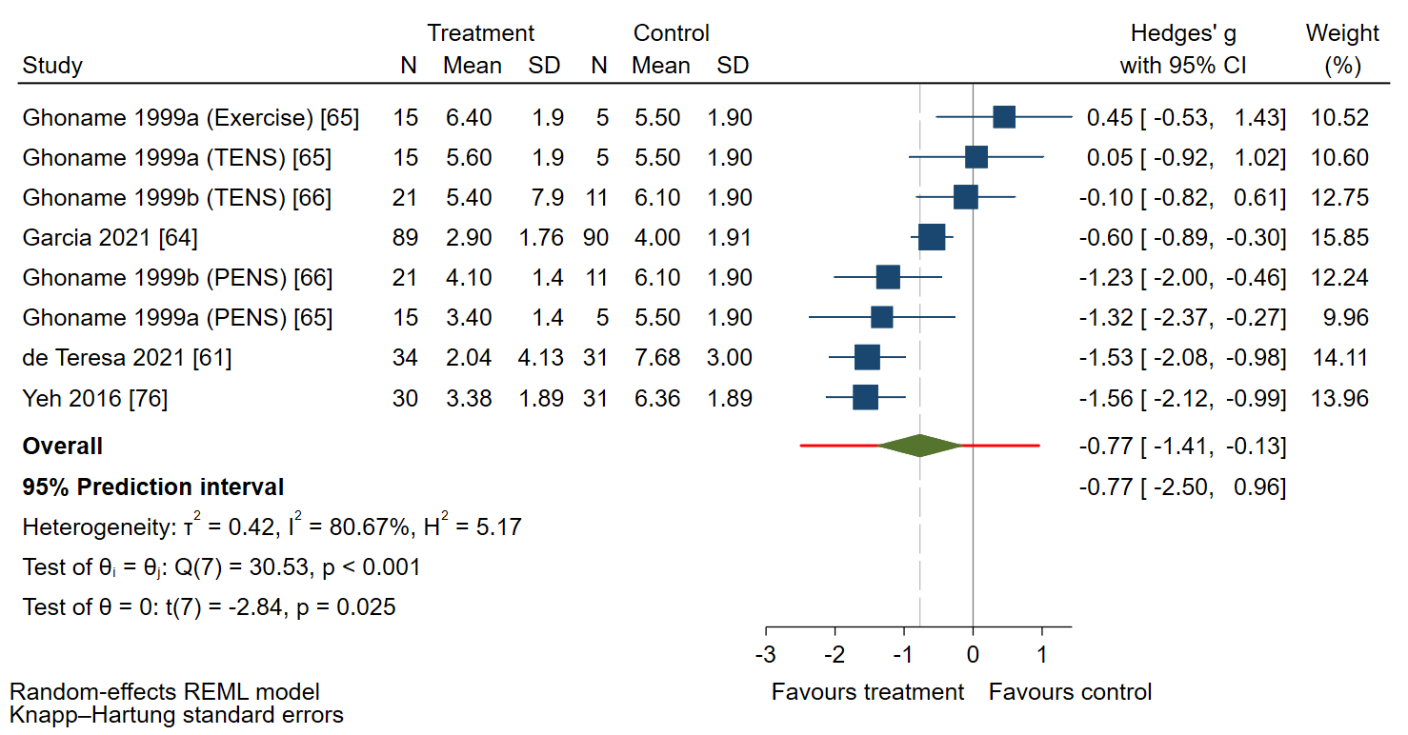

Supplement: Supplementary file 1 [file Table_1.docx]
